# Intersecting experimental evolution and CRISPR screens to identify novel toxin resistance loci

**DOI:** 10.7554/eLife.111773

**Published:** 2026-09-11

**Authors:** Michele Marconcini, Steeve Cruchet, Srishti Goswami, Raghuvir Viswanatha, Matthew Butnaru, Joydeep De, Camilla Roselli, Dafni Hadjieconomou, Norbert Perrimon, Stephanie E Mohr, Richard Benton

**Affiliations:** 1 Center for Integrative Genomics, Faculty of Biology and Medicine, University of Lausanne Lausanne Switzerland; 2 Department of Genetics, Blavatnik Institute, Harvard Medical School Boston United States; 3 Howard Hughes Medical Institute Boston United States; 4 Institut du Cerveau-Paris Brain Institute, Sorbonne Université, Inserm, CNRS Paris France; CNRS - Universite Paris Cite France; New York University United States

**Keywords:** *D. simulans*, *D. sechellia*, S2R+ cells, toxicology, selection, experimental evolution, *D. melanogaster*

## Abstract

Understanding toxin resistance in insects is key to appreciating niche adaptations but remains challenging due to its often-polygenic basis. A well-known example is the specialized association of *Drosophila sechellia* with noni fruit (*Morinda citrifolia*), which is toxic to other insects, including *Drosophila simulans* and *Drosophila melanogaster*. The main noni toxin is octanoic acid (OA), but the mechanisms that determine sensitivity or resistance to OA in different species remain unclear. Here, we experimentally evolved *D. simulans* with increased OA resistance, identifying multiple loci under selection. Cross-referencing these with a genome-wide, OA resistance CRISPR screen in a *D. melanogaster* cell line highlighted two proteins: Kraken, a putative detoxification enzyme expressed in digestive and renal tissues, and Alkbh7, a mitochondrial protein linked to fatty acid metabolism. Both genes show elevated expression in *D. sechellia* and OA-resistant *D. simulans*. In *D. melanogaster*, *kraken* mutants are more OA-sensitive, while *Alkbh7* overexpression increased OA resistance. Mutation of these genes in *D. sechellia* reduced OA tolerance. Our identification of genes contributing to OA resistance in laboratory and natural contexts demonstrates how complementary selection approaches can provide insights into complex mechanisms of toxin susceptibility and adaptation. Such methods could have practical applications in the characterization of natural and artificial insecticides.

## Introduction

How insects resist toxic chemicals in the environment is of both basic scientific interest – reflecting insects’ arms race with plant food sources (Erb and Reymond, 2019; Jones et al., 2022) or venomous predators (Smith et al., 2013) – and applied interest, because, throughout history, humans have used chemicals to combat insect disease vectors and agricultural pests (Umetsu and Shirai, 2020). The best-understood insecticides are those that bind to and modulate neuronal ion channels, where resistance arises through changes in these target proteins (Sparks et al., 2020; Raisch and Raunser, 2023). For example, dichlorodiphenyltrichloroethane (DDT) and pyrethroids prevent closure of voltage-gated sodium channels, resulting in neuronal excitotoxicity and organismal paralysis; diverse insect species displaying resistance to these chemicals have independently arising mutations in genes encoding these channels (Busvine, 1951; Dong, 2007). Similarly, neonicotinoids bind to and hyperactivate nicotinic acetylcholine receptors, leading to neuronal death; mutations in various receptor subunits can confer resistance (Matsuda et al., 2020). Even in such well-studied examples, however, it is thought that other neuronal targets exist (Soderlund and Bloomquist, 1989) and/or that the chemicals impact receptors in non-neuronal tissues (Di Prisco et al., 2013). For many insecticides – as well as other environmental pollutants (Gandara et al., 2024) – the mode of action is incompletely, or not at all, characterized (Sparks et al., 2020).

Resistance observed in nature is only partially understood, reflecting the contribution of many genes beyond those encoding target proteins, which can underlie distinct defense mechanisms (Clarkson et al., 2018; Catteruccia, 2020; Lucas et al., 2023). For example, thickening of the cuticle to reduce entry of insecticides into the body is widely considered an effective resistance mechanism (Balabanidou et al., 2018; Balabanidou et al., 2019). Cuticular changes have been linked to overexpression of proteins implicated in exoskeleton development (e.g. cuticle proteins, ABC transporters), but their precise roles, and how they are upregulated, remain unclear (Balabanidou et al., 2018). Increased expression of metabolic enzymes that degrade insecticides is another commonly observed adaptation in resistant strains, sometimes resulting from copy-number increases in the corresponding genes (Weetman et al., 2018). Duplicated metabolic genes can also undergo neofunctionalization to produce enzymes with altered specificity, as illustrated by Cytochrome P450s in strains of the brown planthopper that recently evolved resistance to the neonicotinoid imidacloprid (Zimmer et al., 2018), as well as by more ancient evolution of glutathione *S*-transferases during the transition of the *Scaptomyza* genus of Drosophilidae to herbivory to counter toxic isothiocyanates (Gloss et al., 2014). However, the lack of experimental resources in many insect species renders it difficult to characterize the causal contribution of specific genetic changes.

**Figure 1.**
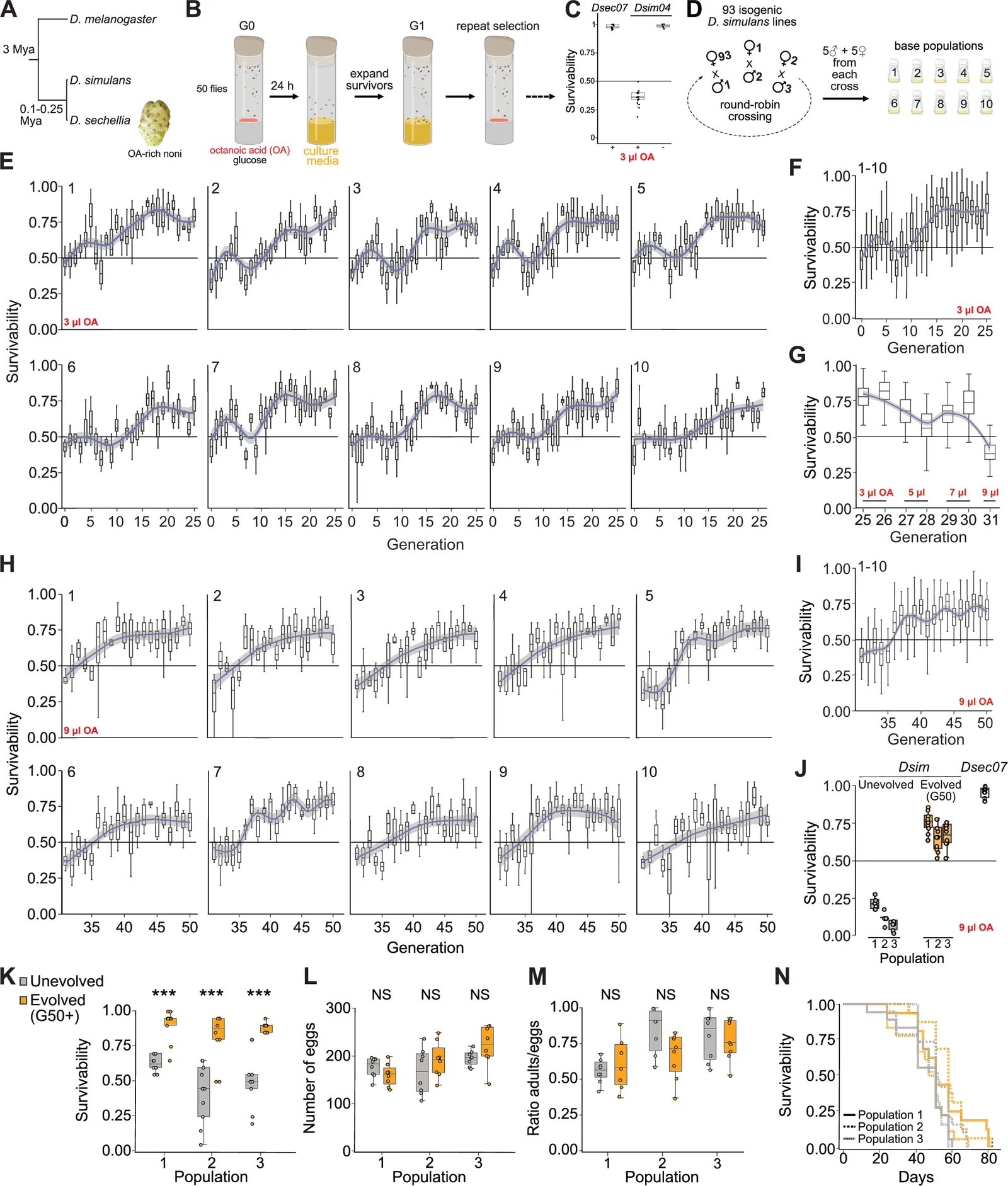
Experimental evolution of *D.* *simulans* with increased octanoic acid (OA) resistance. (**A**) Phylogeny of the drosophilid trio studied in this work. Mya: million years ago. (**B**) Schematic of the OA resistance tube assay (see Materials and methods) and selection procedure. (**C**) Differences in resistance of *D. simulans* 04 and *D. sechellia* 07 strains to 3 μl OA. (**D**) Schematic illustrating the generation of the base populations for Evolve-and-Resequence (see Materials and methods). (**E**) Changes in survivability to 3 μl OA of the 10 *D. simulans* populations over generations 0–25. Box plots represent the interquartile ranges of the survivability for all the replicates; blue lines show the generalized additive model (GAM) regression; gray shading represents the 95% confidence interval for the mean responses at each time point. (**F**) As in **E** for the 10 populations combined. (**G**) OA resistance for the combined 10 populations with increasing OA volumes during generations 25–31. (**H**) As in **E**, with 9 μl OA during generations 31–50. (**I**) As in **H** for the 10 populations combined. (**J**) Comparison of OA resistance of unevolved *D. simulans* (i.e. the original 10 outcrossed populations maintained on standard fly food; see Materials and methods), evolved (generation 50 [G50]) *D. simulans*, and *D. sechellia*. (**K–N**) Phenotypic comparison of unevolved and evolved (G50+) *D. simulans* populations 1–3 for **K** noni resistance (G54), **L** fecundity (G54), **M** developmental viability (G57), and **N** lifespan (G57). For **K–M**, significance was assessed using the unpaired t-test correcting for multiple testing. NS p>0.05, ***p<0.001. For **L–N**, tests were run on standard media. No significant differences between unevolved and evolved flies were detected using the Cox regression model. See Materials and methods for details and Figure 1—source data 1 for raw data. Panel B was created with BioRender.com. Figure 1—source data 1.Octanoic acid resistance and phenotypic characterization of evolved populations.

A compelling natural model clade to study the evolution of toxin resistance is the trio of closely related drosophilid species, *Drosophila melanogaster*, *Drosophila simulans*, and *Drosophila sechellia* (Figure 1A). *D. sechellia* is an extreme specialist on the toxic noni fruit of the *Morinda citrifolia* shrub (Jones, 2005; Auer et al., 2021). Among *D. sechellia*’s numerous adaptations (R’Kha et al., 1991; Dekker et al., 2006; Auer et al., 2020; Auer et al., 2021; Álvarez-Ocaña et al., 2023; Shahandeh et al., 2024), this species has evolved high resistance to octanoic acid (OA), an abundant noni chemical that is toxic to other drosophilids and more divergent insects (Legal et al., 1994; Jones, 1998; Legal et al., 1999; Markow, 2019; Auer et al., 2021; Kaczmarek et al., 2022; Kaczmarek et al., 2024). Previous pioneering studies of *D. sechellia*’s resistance to OA have been studied through genomic mapping-based approaches in *D. sechellia*/*D. simulans* hybrids, which revealed this trait to be polygenic, comprising at least five genomic regions (Amlou et al., 1997; Amlou et al., 1998; Jones, 1998; Huang and Erezyilmaz, 2015). One of the main effect loci encompasses a cluster of 18 genes, including members of the *Osiris* (*Osi*) family, which are thought to function in cuticle patterning (Ando et al., 2019; Sun et al., 2024). RNA interference (RNAi) knockdown of some *Osi* genes in *D. melanogaster* led to reduced tolerance of OA (Andrade López et al., 2017; Lanno et al., 2019). However, genetic evidence for the contribution of these genes to OA resistance in *D. sechellia* is lacking.

Given the challenges of identifying candidate genes through genomic mapping-based strategies, in this work we took two complementary approaches to investigate the evolution of OA resistance in this drosophilid trio: experimental evolution in *D. sechellia*’s closest relative, *D. simulans*, through an Evolve-and-Resequence strategy (Schlötterer et al., 2016), and CRISPR/Cas9-based genome-wide genetic screening in cultured *D. melanogaster* cells (Viswanatha et al., 2018). We show how the intersection of these approaches can identify novel genes contributing to OA susceptibility/resistance in these drosophilids.

## Results

### Experimental evolution of OA resistance in *D. simulans*

A previous study demonstrated that *D. simulans* strains can be experimentally evolved for higher OA resistance, enabling the identification of genomic regions (through microsatellite markers) responding to selection (Colson, 2004). This success inspired us to take an Evolve-and-Resequence approach, which uses high-throughput sequencing to analyze the entire genome of populations before and after exposure to specific selective pressures, thereby facilitating the identification of adaptive loci from standing genetic variation in controlled and replicable settings (Turner and Miller, 2012; Schlötterer et al., 2016; Barghi et al., 2019; Kelly and Hughes, 2019; Shahrestani et al., 2021). For studies of insecticide resistance, it has been predominantly used to examine changes in frequency of alleles of known genes rather than as a means to identify novel loci (Zoh et al., 2021; Sadia et al., 2024).

The susceptibility/resistance of drosophilids to OA can be easily assessed by exposing flies to the toxin in a culture tube and counting the survivors after 24 hr (Figure 1B). In line with previous observations (Amlou et al., 1997; Colson, 2004), we found that a dose of 3 μl of pure OA (mixed with 1 ml glucose solution), corresponding to roughly half the amount estimated in 2 g of noni pulp (see Materials and methods), leads to death of ~60% of *D. simulans* but has no effect on *D. sechellia* (Figure 1C). By collecting the *D. simulans* survivors, we reasoned that we could allow these to mate and establish the next generation (Figure 1B), thus enriching genetic variants conferring higher resistance, similarly to Colson, 2004. Repeating the process over multiple generations and multiple replicates would allow us to track phenotypic changes in resistance and investigate the associated trajectories in allele frequencies.

Standing genetic variation should be present in the evolving populations for natural selection to act upon (Teotónio et al., 2009; Long et al., 2015). As the spontaneous mutation rate in drosophilids is too low for artificial selection (Keightley et al., 2014), we generated 10 independent polymorphic base populations of *D. simulans* from 93 isogenic strains (Signor et al., 2018) by round-robin crossing (Figure 1D). These lines contain millions of SNPs, but we note that they were sampled from a single wild population in California; therefore, reflecting the genetic variation present in that locality. The base populations therefore might not include all variants found across the species’ range, including those in regions where *D. sechellia* co-occurs. To minimize bottleneck effects, each population was kept at a size of ~1000 flies. At each generation, ~500 flies from each population were randomly collected and selected for resistance to 3 μl OA. Over the course of 25 generations, all the populations showed increased levels of survivability, suggesting parallel adaptive evolution (Figure 1E). Given the consistent responses across all populations, we also analyzed the phenotypic changes grouping all replicates, finding that the mean survivability significantly increased from ~43% at generation 0 to ~78% at generation 25 (Figure 1F).

As we kept the same amount of OA throughout the 25 generations of selection, the selective pressure lowered as the populations’ survivability increased, evidenced as a larger fraction of flies surviving and reproducing. Viewing the phenotype of populations both individually (Figure 1E) and collectively (Figure 1F), it was apparent that the change in phenotype plateaued at around generation 15. For continued evolution, we therefore gradually increased the amount of OA during generations 26–31 (Figure 1G), reaching a selection pressure comparable to the initial phase of the experiment with 9 μl OA (~38% mean survivability across all populations) (Figure 1G). We then performed a second round of selection at this dose, observing an increase in survivability across all populations, reaching ~70% by generation 50 (Figure 1H). As the populations adapted to the new condition, a second plateau started around generation 40 (Figure 1H and I). Over 50 generations we were therefore able to evolve *D. simulans* to have approximately fivefold higher survivability to 9 μl OA compared to the unevolved populations (Figure 1J), though still falling short of the survival exhibited by *D. sechellia* (Figure 1J). These plateaus can be explained by the decreasing selective pressure exerted by fixed OA volumes as an ever-greater proportion of flies adapt and survive the treatment. Determining whether an upper limit exists that would constrain the evolution of a *D. sechellia*-like level of phenotypic resistance would require additional selection rounds using progressively higher OA concentrations over an extended (and unknown) number of generations. OA is only one component of noni fruit, although probably the major toxic chemical (Legal et al., 1994). We therefore tested three of our evolved populations for resistance to noni fruit pulp, finding that they exhibited much higher survival on this substrate compared to the same original populations maintained under neutral conditions (i.e. no OA selection) (Figure 1K).

Specialization of *D. sechellia* on toxic noni is associated with a reduced egg-laying capacity (R’ kha et al., 1997; Markow et al., 2009) and a shorter (albeit strain-specific) lifespan (Watada et al., 2020; Abe et al., 2022; Shahandeh et al., 2024). Our success in evolving *D. simulans* with a partial *D. sechellia*-like resistance trait begs the question as to whether these selected strains exhibit other phenotypic changes as a trade-off for higher toxin resistance. We tested whether the evolved resistant populations of *D. simulans* exhibit similar phenotypic changes in control conditions. However, none of the three focal populations tested exhibited significant decreases in the number of eggs laid (Figure 1L), developmental viability (Figure 1M), or lifespan (Figure 1N). Thus, at least within the timeframe of our experimental evolution experiment, *D. simulans* did not display obvious phenotypic trade-offs for the gain in OA resistance.

### Dynamic directional selection of allele frequencies

**Figure 2.**
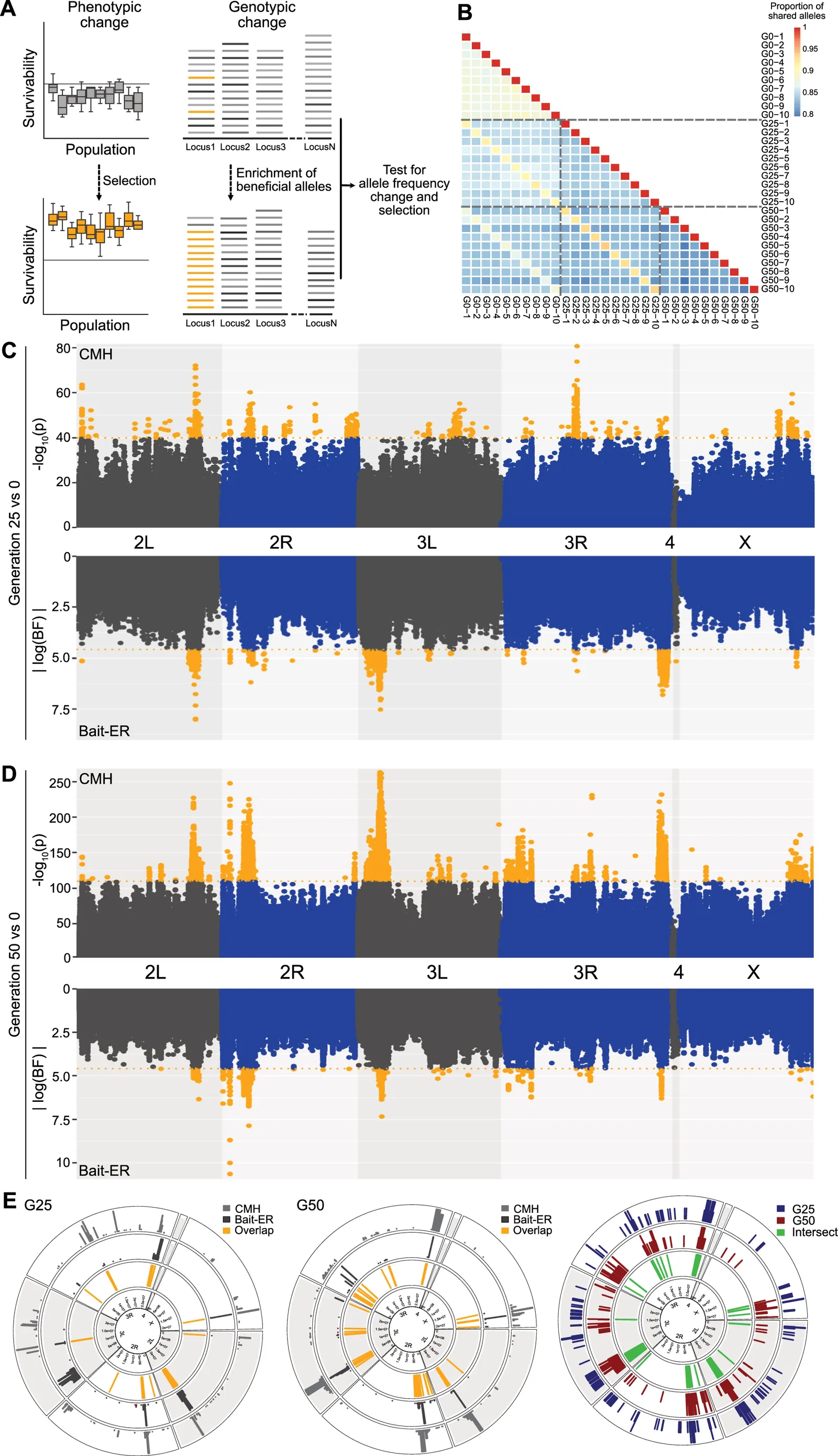
Dynamic directional selection of allele frequencies during experimental evolution. (**A**) Schematic of the analysis. (**B**) Identity-by-state analysis. The heatmap depicts the proportion of shared alleles between populations, with values ranging from 0.8 (blue, lowest shared proportion) to 1 (red, identical alleles). (**C,D**) Manhattan plots showing the probability of allele frequency changes between (**C**) generations 0 and 25, and (**D**) generations 0 and 50. Top panel shows the results of the Cochran-Mantel-Haenszel (CMH) test; orange dots indicate significant variants at a threshold of –log_10_(p)≥40, a conservative approximation of the top 0.000005% simulated SNPs under neutrality (Figure 2—figure supplement 1). Bottom panel shows the absolute log-Bayes factors estimated by Bait-ER; orange dots indicate significant variants at the conservative threshold log(0.99/0.01). (**E**) Circular plots illustrating overlap of predicted genomic regions under directional selection between the two statistical approaches at generation 25 (left) and generation 50 (middle), or between generations 25 and 50 (right). Raw data in Figure 2—source data 1. Figure 2—source data 1.Selected genomic intervals and associated genes.

We hypothesized that the observed phenotypic changes in *D. simulans* depended on the directional selection of alleles of consistent loci across the 10 populations, while most of the genome evolves under neutrality. Allele frequencies at the selected loci should therefore consistently change across all populations, allowing us to statistically distinguish them from randomly evolving loci (Figure 2A). We sequenced the genomes of the populations of evolved (generation 25 and generation 50) and unevolved (generation 0) flies, identifying 5,127,840 high-quality variants distributed across all chromosomes. Pairwise identity-by-state (IBS) analysis indicated that as the number of generations increased, the proportions of shared alleles decreased, both between time points and between pairs of populations (left-to-right and top-to-bottom, respectively, in Figure 2B).

**Figure 2—figure supplement 1.**
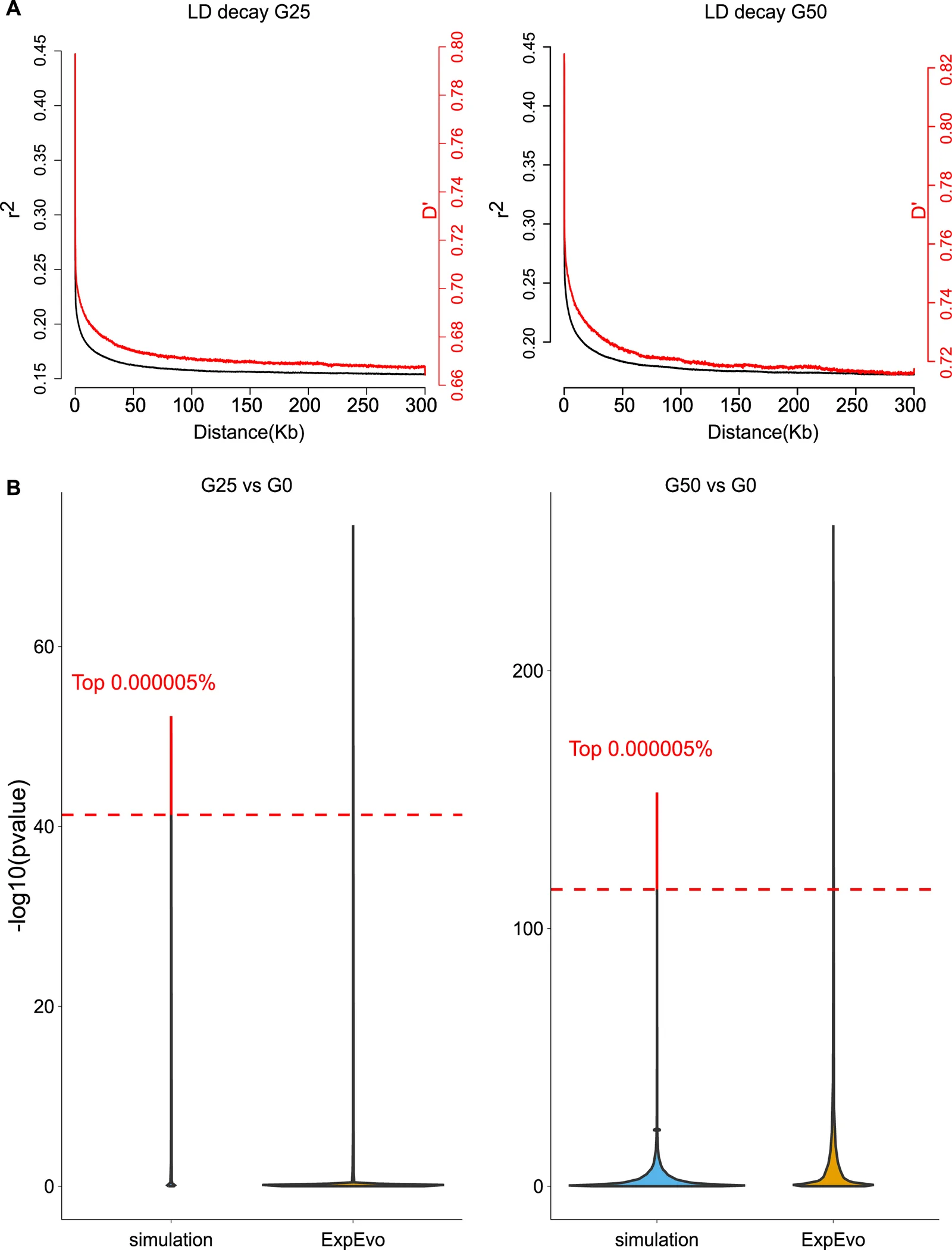
Linkage disequilibrium decay, and comparison of simulated and observed p-value distributions in the experimentally evolved populations. (**A**) Linkage disequilibrium decay at generations 25 (left) and 50 (right). Black curves represent the trajectory of r2 values (y-axis left) as genomic distances increase (x-axis). Red curves represent the same but for D' values (y-axis right). (**B**) Violin plots showing the distribution of p-values from the Cochran-Mantel-Haenszel (CMH) test, comparing allele frequency changes between generations 25 and 0 (left), and generations 50 and 0 (right). Blue violins represent p-values derived from forward evolution simulations, while orange violins correspond to p-values from experimentally evolved populations. Red dashed lines mark the top 0.000005% of simulated p-values, serving as the significance threshold for the experimental data.

To identify the selected genetic variants underlying the phenotypic adaptation, we analyzed the genome-wide allele frequency changes both between generations 0 and 25, and between generations 0 and 50, across all populations. The identification of significant changes in frequency is non-trivial, and many methods have been developed (Vlachos et al., 2019; Kojima et al., 2020; Barata et al., 2023). We therefore assessed the significance of frequency changes using two complementary approaches: the Cochran-Mantel-Haenszel (CMH) test (Cochran, 1954; Mantel and Haenszel, 1959), a widely used method that tests for allele frequency changes between two time points, and Bait-ER (Barata et al., 2023), a Bayesian method that tests for selection while explicitly modeling genetic drift. Both tests identified large portions of the genome under positive selection at generations 25 and 50 (Figure 2C and D). After 25 generations, the two methods shared 1.90 Mb of candidate regions; the overlap encompassed 65.3% of the genomic span identified by CMH and 64.1% of the span identified by Bait-ER. After 50 generations, the two methods shared 4.77 Mb of candidate regions; here, the overlap encompassed 63.6% and 99.9% of the CMH and Bait-ER candidate regions, respectively (Figure 2E).

Based on the analysis of genome-wide linkage disequilibrium decay (Figure 2—figure supplement 1), we grouped the significant SNPs identified by each of the two methods into genomic blocks according to their proximity (i.e. <50 kb). We retrieved all the candidate genes that fall within the block intervals, obtaining a total of 439 and 401 genes for the CMH and Bait-ER analysis, respectively, at generation 25, and 1238 and 174 genes at generation 50 (Figure 2—source data 1). These genes likely include true targets of selection, but also neighboring genes affected by hitchhiking. The increase in CMH candidate genes over time – in contrast to the decrease observed with Bait-ER – might reflect the CMH test’s tendency to overestimate significance and to be insensitive to divergence among replicate populations.

The overlap of all significant genes between the two time points is <30%. This might reflect that a large fraction of the candidate genes results from noise caused by genetic drift and draft (hitchhiking) and/or reflect a shift in the phenotypic contribution of genes under the stronger selection (i.e. the higher dose of OA) during generations 27–50. Moreover, previous work on *Drosophila* using temporal sampling showed that adaptation involving complex traits can lead to non-overlapping sets of selected SNPs, in contrast with the simple expectation of continuous increases in allele frequency until fixation (Orozco-terWengel et al., 2012; Schlötterer et al., 2016). Gene ontology analysis is complicated at this stage as most hits are likely hitchhikers of the true causal genes. It is also hard to compare currently to previous mapping studies in *D. simulans*/*D. sechellia* hybrids (Amlou et al., 1997; Amlou et al., 1998; Huang and Erezyilmaz, 2015) or in the earlier *D. simulans* experimental evolution experiment (Colson, 2004), which only identified wide genomic regions. The sole exception, a 170 kb window on 3R containing only 18 genes (including several *Osiris* genes) (Hungate et al., 2013), did not emerge in our analysis.

### A genome-wide, pooled CRISPR screen for OA susceptibility and resistance genes in *D. melanogaster* S2R+ cells

**Figure 3.**
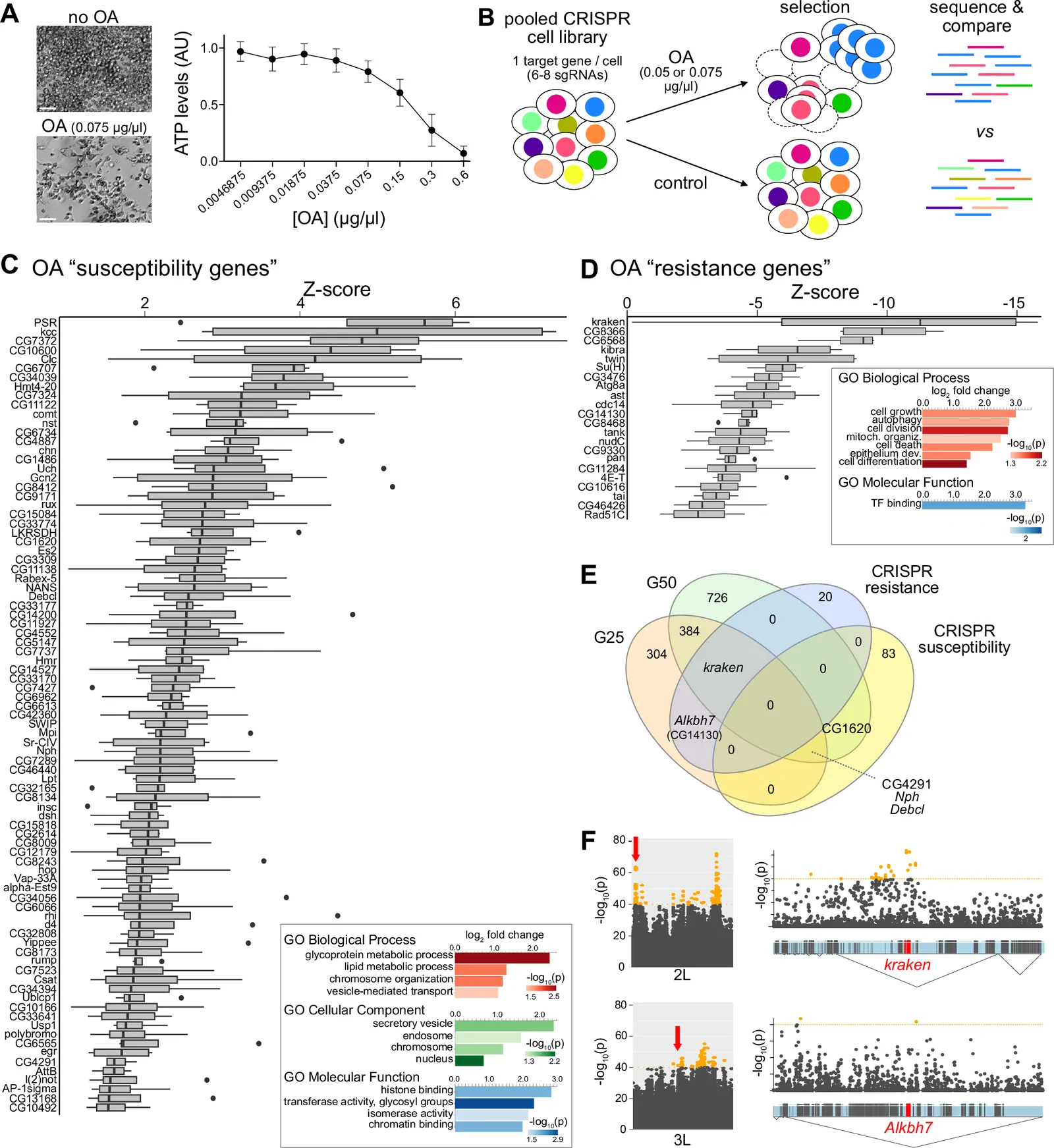
A genome-wide pooled CRISPR screen for octanoic acid (OA) susceptibility and resistance genes in *D.* *melanogaster* cultured cells. (**A**) Left: images of S2R+ cells cultured in the absence or presence of OA. Scale bar, 50 μm. Right: dose-dependent effects of OA on S2R+ cell viability, through measurement of ATP levels with a CellTiter-Glo assay (see Materials and methods). (**B**) Schematic of CRISPR screen design. (**C**) Top 5% ‘susceptibility’ genes (see Materials and methods and Figure 3—source data 1). Box plots represent the interquartile ranges for all the replicates of the Z-scores obtained through maximum likelihood estimation to test for selection. The inset (bottom-right) displays the gene ontology analysis of these genes. (**D**) As in **C**, for the top 5% ‘resistance’ genes. (**E**) Venn diagram showing the overlap between all the genes identified in the experimental evolution at G25 and G50, and genes associated with susceptibility and resistance in the CRISPR screen. (**F**) Subportion of the top panel (Cochran–Mantel–Haenszel [CMH] test) of the Manhattan plot from Figure 2C for chromosome arms 2L and 3L, with the red arrows highlighting the regions magnified on the right showing the significant variants and linkage disequilibrium blocks (triangles) for *kraken* and *Alkbh7* (CG14130). Figure 3—source data 1.Results of the genome-wide pooled CRISPR screen and PANGEA analysis.

Given that the previous mapping studies also contain extensive hitchhiking signals and substantial noise across large genomic regions, comparing their results with our analyses would likely yield numerous false positives and still leave a large pool of putative loci, offering limited benefit for homing in on candidate genes. We therefore sought an orthogonal screening approach. Motivated by previous observations of OA toxicity for insect Sf9 cultured cells (Kaczmarek et al., 2022), we found that OA also leads to reduced viability of *D. melanogaster* S2R+ cells (Figure 3A). This phenotype prompted us to perform a selection experiment in this cell line through exploitation of a genome-wide, pooled CRISPR/Cas9 mutagenesis approach (Figure 3B; Viswanatha et al., 2018). In brief, a library of sgRNA-encoding plasmids (6–8 sgRNAs/gene) was transfected into Cas9-expressing cells where site-specific plasmid integration predominantly limits targeting to a single gene per cell. Two replicate cultures of this pool of cells were either left untreated or exposed to OA in the medium, at two different concentrations (Figure 3B). After 26 days in culture, DNA was extracted from the cell pool, then sgRNA sequences were PCR-amplified and sequenced to determine those that were enriched after OA selection (reflecting genes whose loss enhances cell viability, referred to hereafter as ‘susceptibility genes’) or depleted (reflecting genes whose loss leads to lower OA resistance, hereafter ‘resistance genes’).

To identify candidate OA susceptibility/resistance genes, we first filtered out genes essential for general cell survival (Viswanatha et al., 2018) and genes that were not detectably expressed in S2R+ cells (using DGET; Hu et al., 2017). (Removal of such genes are inevitable limitations of this cell-based screening approach; see Discussion.) After comparing results from each condition and replicate, we identified the top 5% susceptibility genes (Figure 3C) and top 5% resistance genes (Figure 3D). Among the 87 susceptibility genes, there was no single stand-out hit, suggesting that – at least in S2R+ cells – OA does not have a single major cellular target (unlike, for example, insecticidal bacterial toxins that bind a specific cell surface receptor; Xu et al., 2022). Gene ontology analysis of these susceptibility genes revealed a significant enrichment of genes involved in membrane trafficking (Figure 3C). As OA has previously been hypothesized to disrupt cellular membranes (Borrull et al., 2015; Kaczmarek et al., 2022), the recovery of these hits suggests that general reduction in membrane transport reduces OA uptake into cells and/or renders membranes less susceptible to OA treatment. 22 OA resistance genes were identified, encoding diverse types of proteins (Figure 3D), suggesting that there are multiple mechanisms by which cells can resist the toxic effects of OA.

We next examined which of the genes identified in the S2R+ cell selection experiments might be relevant for evolution of OA resistance in whole animals by determining those also present within significant blocks of the experimental evolution analyses (Figure 3E and F). Four susceptibility genes were identified: CG1620 (a predicted SANT-MYB domain transcription factor), CG4291 (a putative component of the splicing machinery), *Nph* (a chromatin remodeling factor; Emelyanov et al., 2014), and *Debcl* (a pro-apoptotic member of the Bcl-2 family; Galindo et al., 2009); these are all likely to have indirect roles in conferring susceptibility to OA given their broad functions. By contrast, two resistance genes have potentially direct roles in conferring tolerance to OA: *kraken* and CG14130. *kraken* encodes a putative serine hydrolase (Edwin Chan et al., 1998) and stood out both because it was the top hit in the resistance gene screen (Figure 3D), and because of its location in one of the most prominent peaks in the experimental evolution at generations 25 and 50 (Figures 2C, D, 3F). CG14130 encodes the orthologue of mammalian Alkbh7, which is involved in regulating fatty acid metabolism (Solberg et al., 2013; Zhang et al., 2021), and is located within a significant peak at generation 25 (Figures 2C and 3F). Therefore, intersecting the experimental evolution and CRISPR screening results allowed us to narrow down hundreds of potential candidates to two focal genes for downstream investigations.

### Sequence, expression, and evolutionary analyses of candidate OA resistance genes

**Figure 4.**
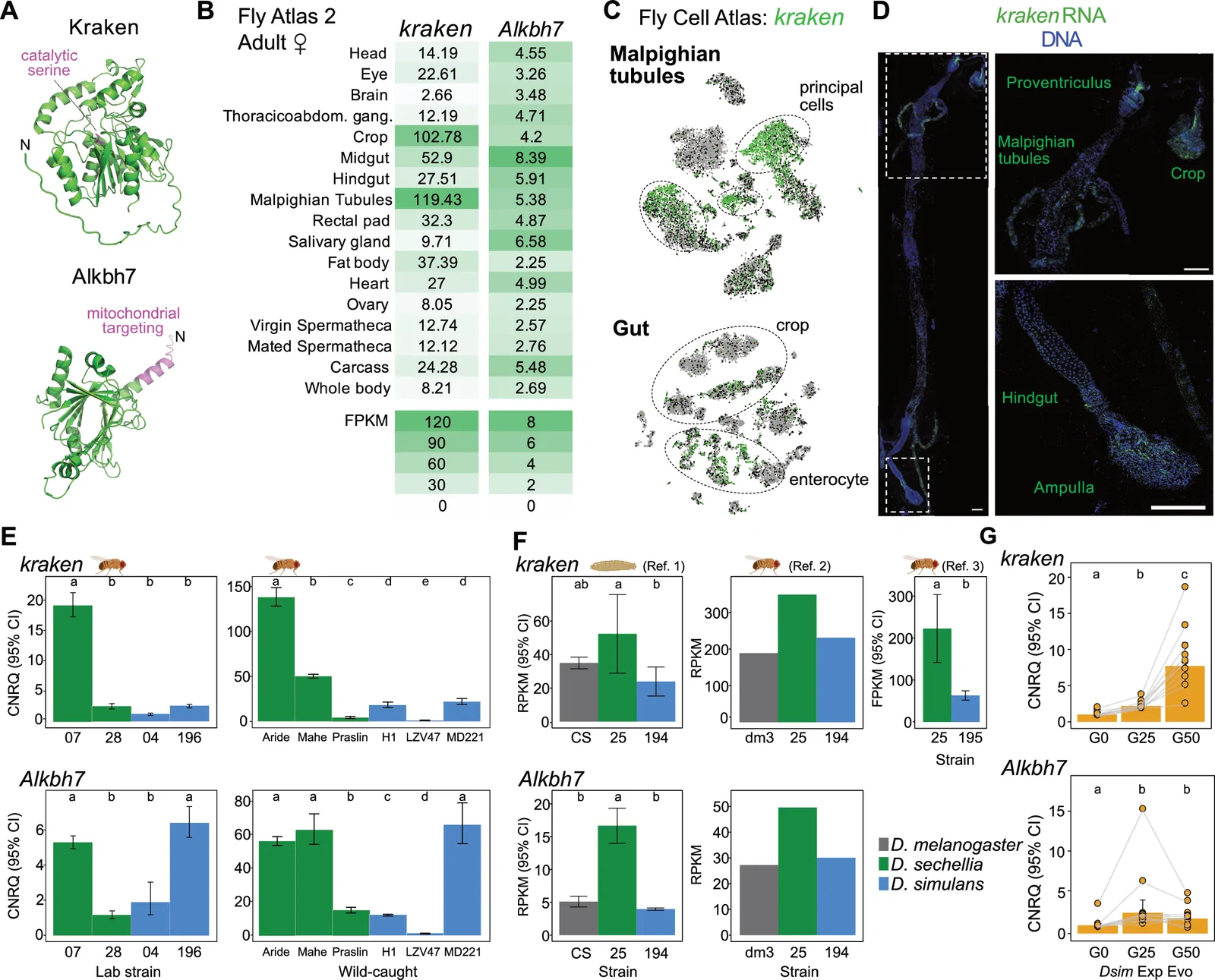
Sequence and expression analyses of *kraken* and *Alkbh7*. (**A**) AlphaFold protein models for *D. melanogaster* Kraken (AF-O18391-F1-model_v4) and Alkbh7 (AF-Q9VTP1-F1-model_v4) (Jumper et al., 2021; Varadi et al., 2022), highlighting a conserved putative catalytic serine in the active site in Kraken, and the mitochondrial targeting sequence in Alkbh7 (predicted by MitoFates; Fukasawa et al., 2015). See also Figure 4—figure supplement 1. (**B**) Tissue-specific expression of *D. melanogaster kraken* and *Alkbh7* from bulk RNA-sequencing data from the Fly Atlas 2.0 (Krause et al., 2022). (**C**) tSNE plots illustrating *D. melanogaster kraken* expression in single-cell transcriptomes of the Malpighian tubules and gut from the Fly Cell Atlas (Stringent 10× datasets) (Li et al., 2022). (**D**) Left: RNAscope detection of *kraken* (green) transcripts in the gut and Malpighian tubules, with nuclei counterstained with DAPI (blue). Right: higher-magnification images showing *kraken* transcript expression in the indicated tissues. Scale bars, 200 μm. This expression pattern was observed in tissues from >20 individuals. (**E**) Expression levels of the *kraken* and *Alkbh7* in the indicated laboratory strains (left panel) and wild-caught strains (right panel) (Shahandeh et al., 2024; Shahandeh et al., 2026) of adult female *D. sechellia* and *D. simulans* measured by quantitative PCR (qPCR) (Figure 4—source data 1). Expression is represented as calibrated and normalized relative quantities (CNRQ). Significance was assessed using the unpaired t-test correcting for multiple testing and represented using letter codes. (**F**) Expression levels of *kraken* and *Alkbh7* in the indicated strains and species at larval or adult stages as indicated from published RNA-sequencing data (References: 1, Watanabe et al., 2019; 2 Ma et al., 2018; 3 Kalra et al., 2024). Significance was assessed using the unpaired t-test correcting for multiple testing and represented using letter codes when multiple replicates were present in the original data. (**G**) Expression levels of the candidate genes at generations 0, 25, and 50 of the experimentally evolved *D. simulans* populations measured by qPCR (Figure 4—source data 1). Significance was assessed using the paired t-test correcting for multiple testing and represented using letter codes. Panels E and F were created with BioRender.com. Figure 4—source data 1.Quantitative PCR (qPCR) expression of *kraken* and *Alkbh7* in different strains.

To assess the candidacy of *kraken* and CG14130 (hereafter *Alkbh7*) in contributing to OA resistance in animals, we first examined their sequence and expression in *D. melanogaster*. Kraken belongs to a large family of serine hydrolases, which encompass lipases, esterases, and proteases that use a nucleophilic serine residue in the active site for hydrolysis of substrates (Edwin Chan et al., 1998). Consistent with the conservation of this serine in Kraken (Figure 4A, Figure 4—figure supplement 1), an activity-based proteomic screen of the serine hydrolase superfamily in *D. melanogaster* provided evidence for in vivo catalytic activity of Kraken (Kumar et al., 2021). *kraken* is broadly expressed in adults, with particular enrichment in the gut and Malpighian tubules (Figure 4B), similar to previous observations of *kraken* expression in the embryo by RNA in situ hybridization (Edwin Chan et al., 1998). Using the Fly Cell Atlas (Li et al., 2022), we examined sub-tissue adult expression of *kraken*: in the Malpighian tubules, *kraken* is expressed at highest levels in the principal cells (Figure 4C), which are involved in ion transport and detoxification. In the gut, *kraken* transcripts are most robustly detected in various types of enterocytes, which secrete digestive enzymes (Lemaitre and Miguel-Aliaga, 2013; Figure 4C). However, Kraken lacks an N-terminal signal sequence (Teufel et al., 2022), suggesting that the protein remains in the cytoplasm of these cells, unlike canonical digestive enzymes (Lemaitre and Miguel-Aliaga, 2013), and more similar to detoxification enzymes of the Cytochrome P450 and glutathione *S*-transferase families (Yang et al., 2007). To examine *kraken* expression in vivo, we performed RNA fluorescence in situ hybridization (FISH), which detected *kraken* transcripts in various regions of the gut (crop, crop duct, proventriculus, hindgut, ampulla) and Malpighian tubules (Figure 4D).

**Figure 4—figure supplement 1.**
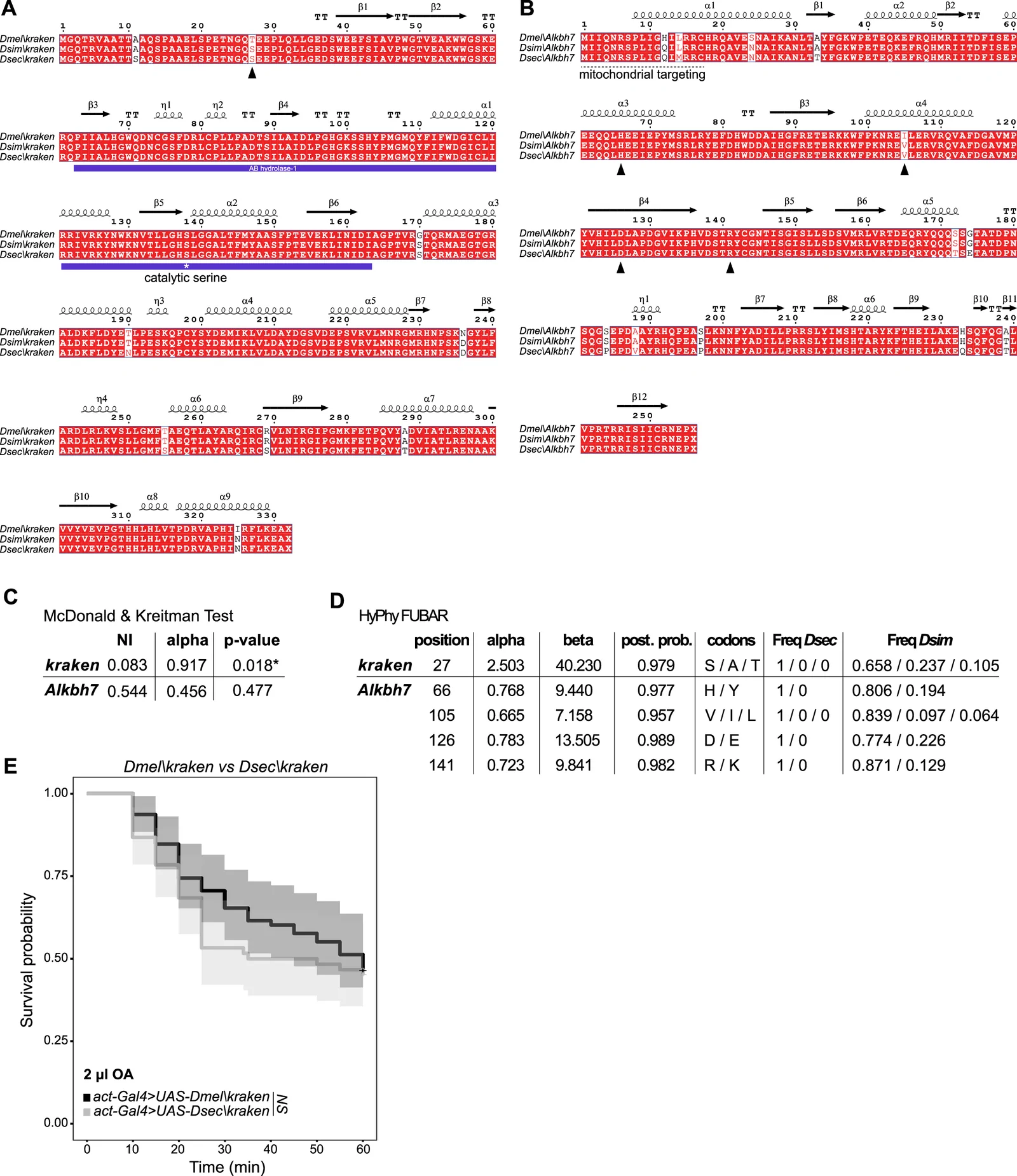
Sequence and molecular evolutionary analyses of *kraken* and *Alkbh7*. (**A,B**) Protein sequence alignment and secondary structure of Kraken (**A**) and Alkbh7 (**B**) from the reference sequence of *D. melanogaster* (NP_477265.1 and NP_648511.2) and the consensus sequences of *D. simulans* and *D. sechellia*. Sequence data from PRJNA215932 and PRJNA395473 (see Materials and methods). Black triangles indicate the residues where signs of selection were detected by the FUBAR (Fast, Unconstrained Bayesian AppRoximation) analysis (see **D**). (**C**) Results from the McDonald-Kreitman test. NI = neutrality index; alpha = proportion of substitutions driven by positive selection; p-value = p-value resulting from a two-tailed Fisher’s exact test. (**D**) FUBAR analysis to detect pervasive selection as implemented in HyPhy. position = codon position; alpha = mean posterior synonymous substitution rate; beta = mean posterior nonsynonymous substitution rate; posterior prob = posterior probability of positive selection; codons = alternative codons, Freq Dsec = frequency of the alternative codons in *D. sechellia* in the same order as codons; Freq Dsim = frequency of the alternative codons in *D. simulans* in the same order as codons. (**E**) Survival curves of *D. melanogaster* overexpressing *Dmel\kraken* and *Dsec\kraken* exposed to 2 μl OA. Each replicate consisted of ten 2- to 7-day-old female flies. Genotypes: *act-Gal4/+;UAS-Dmel\kraken/+*, *act-Gal4/+;UAS-Dsec\kraken/+*. N replicates ≥6, n flies ≥60. Shading indicates 95% confidence intervals. Significance is based on the resulting mixed-effects Cox regression model. NS p>0.05. Raw data in Figure 4—figure supplement 1—source data 1. Figure 4—figure supplement 1—source data 1.Octanoic acid survival of *D. melanogaster* and *D. sechellia kraken* UAS-expressing flies and alignment of *kraken* and *Alkbh7* Coding sequences.

**Figure 4—figure supplement 2.**
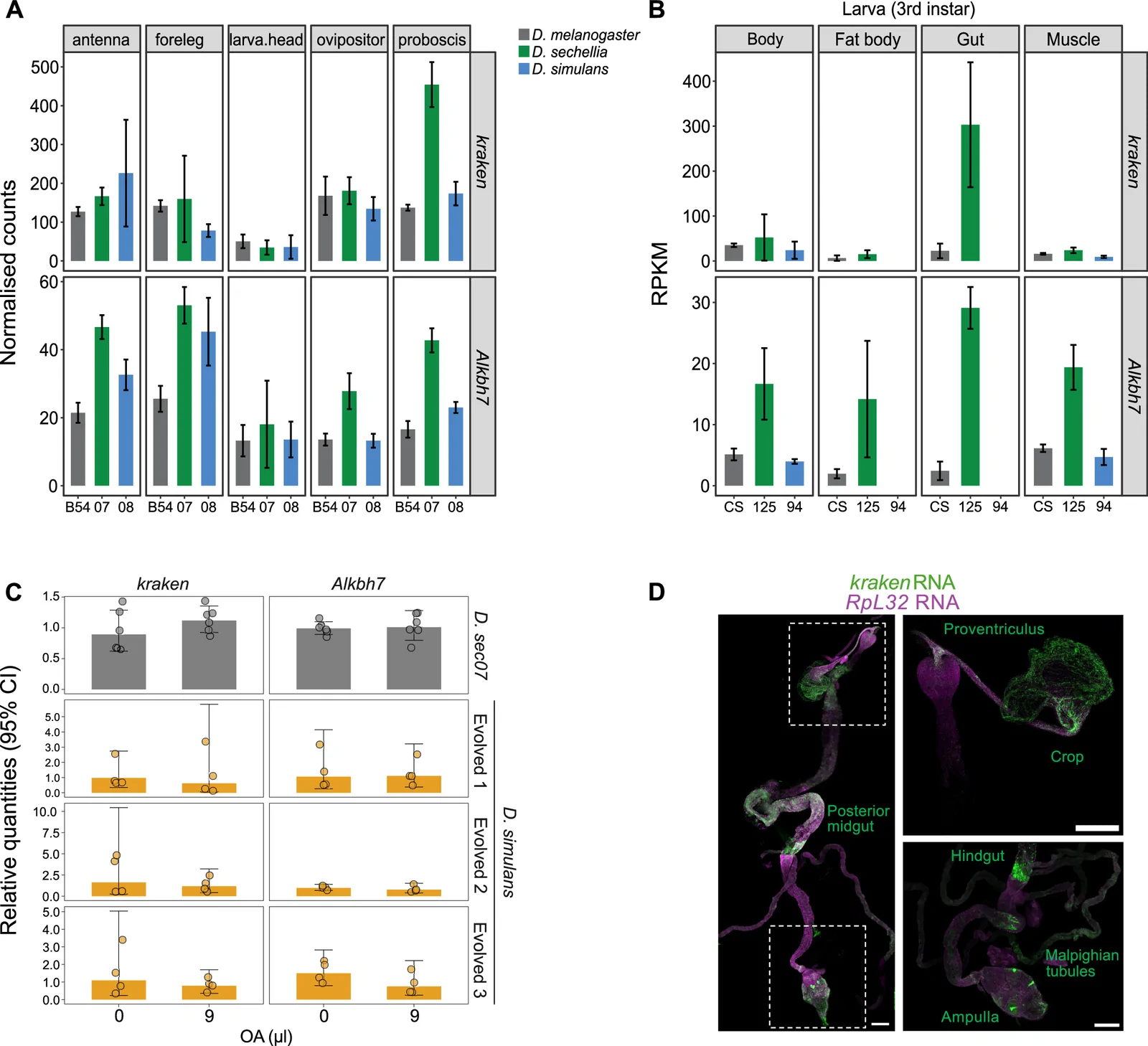
Additional expression analyses of *kraken* and *Alkbh7*. (**A**) Bar plots showing the expression levels of *kraken* and *Alkbh7* across various adult *Drosophila* tissues and species. Data from Bontonou et al., 2024. (**B**) Bar plots showing the expression levels of *kraken* and *Alkbh7* across various larval *Drosophila* tissues and species. Data from Watanabe et al., 2019 (‘medium diet’ condition). (**C**) Bar plots showing the effect of exposure to 9 μl octanoic acid (OA) (in the tube assay) on *kraken* and *Alkbh7* expression in *D. sechellia* and three evolved (**G50**) *D. simulans* populations. (**D**) Left: RNAscope detection of *kraken* (green) and *Rpl32* (magenta) transcripts in the gut and Malpighian tubules. Right: higher-magnification showing *kraken* transcript expression in the indicated tissues. Scale bars, 200 μm. This expression pattern was observed in tissues from >10 individuals.

Alkbh7 is a member of the alpha-ketoglutarate-dependent hydroxylase (Alkbh) family (Figure 4A), which comprises enzymes that act on diverse substrates in processes such as biosynthesis, metabolism, and epigenetic regulation (Xu et al., 2021). As in mammals (Solberg et al., 2013), *D. melanogaster Alkbh7* displays broad tissue expression (Figure 4B). Murine Alkbh7 is localized to the mitochondrial matrix (Solberg et al., 2013). This property appears to be conserved in the *Drosophila* protein, which has a mitochondrial targeting sequence (Figure 4A, Figure 4—figure supplement 1; Fukasawa et al., 2015); consistently, we identified Alkbh7 within *D. melanogaster* proteomes of the mitochondrial matrix (Chen et al., 2015; Sen and Cox, 2022).

Although the sequences of both Kraken and Alkbh7 are overall highly similar across *D. melanogaster*, *D. simulans*, and *D. sechellia* (Figure 4—figure supplement 1), we wondered whether we could detect signs of selection at these loci in *D. sechellia* employing population sequence datasets of *D. sechellia* and *D. simulans* (Schrider et al., 2018). Using the McDonald-Kreitman test (McDonald and Kreitman, 1991) on the coding sequences of both genes, we found evidence for gene-level positive selection on *kraken* but not *Alkbh7* (Figure 4—figure supplement 1). Because selection might act pervasively at individual sites rather than along the entire protein coding region, we also implemented a codon-based analysis using FUBAR (Fast, Unconstrained Bayesian AppRoximation) (Murrell et al., 2013), which identified 1 and 4 codons under diversifying positive selection in *kraken* and *Alkbh7*, respectively (Figure 4—figure supplement 1). Although their functional relevance remains unclear, such mutations might reflect underlying evolutionary dynamics at the protein level.

We next compared the expression levels of these genes both in laboratory and wild-caught strains of *D. sechellia* and *D. simulans* (Figure 4E), as well as in RNA-sequencing datasets of various strains of *D. sechellia*, *D. simulans*, and *D. melanogaster* (Ma et al., 2018; Watanabe et al., 2019; Kalra et al., 2024; Figure 4F, Figure 4—figure supplement 2). While there were some exceptions, overall both *kraken* and *Alkbh7* show higher expression levels in *D. sechellia* adults and larvae compared to the other species. Importantly, in our experimentally evolved *D. simulans* populations, we also found that both *kraken* and *Alkbh7* were more highly expressed compared to unselected populations (Figure 4G). The expression differences between species, and between unevolved and evolved *D. simulans*, reflect variation in basal levels of expression, as prior exposure to OA did not lead to changes in expression in either *D. sechellia* or *D. simulans* (Figure 4—figure supplement 2). RNA FISH of *kraken* in *D. sechellia* revealed similar spatial expression as in *D. melanogaster*, with the addition of a robust signal in the midgut (Figure 4—figure supplement 2). These observations suggest that elevated expression in this species is due, at least in part, to a novel expression pattern in this digestive organ.

### Functional contributions of *kraken* and *Alkbh7* to OA resistance

**Figure 5.**
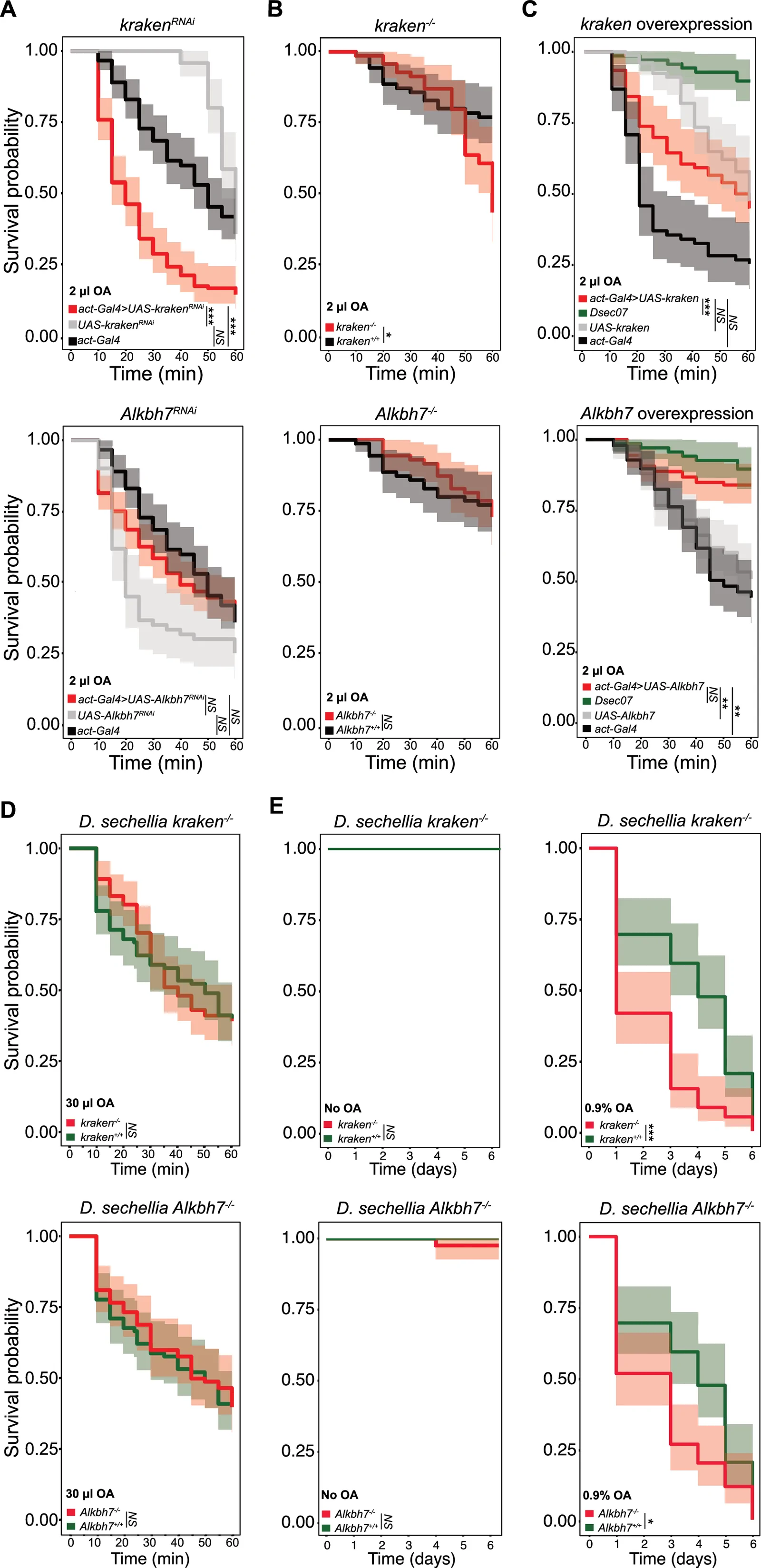
Functional validation of the contribution of *kraken* and *Alkbh7* to octanoic acid (OA) resistance. (**A–C**) Survival curves of adult flies of the indicated genotypes tested in the plate assay with 2 μl OA for *kraken* and *Alkbh7* RNA interference (RNAi) (**A**), mutants (**B**), and overexpression (**C**). Each replicate consisted of ten 2- to 7-day-old female flies. N replicates ≥6, n flies ≥60. Shading indicates 95% confidence intervals. Genotypes: **A***act-Gal4/+;UAS-kraken^RNAi^/+, UAS-kraken^RNAi^/+, act-Gal4/+, act-Gal4/+;UAS-Alkbh7^RNAi^/+, UAS-Alkbh7^RNAi^/+, act-Gal4/+*, **B** *kraken^1^*, *Alkbh7^1^, Canton-S* (wild-type genetic background control for both mutants), **C** *act-Gal4/+;UAS-kraken^o/e^/+*, *UAS-kraken^o/e^/+*, *act-Gal4/+*, *act-Gal4/+;UAS-Alkbh7^o/e^/+*, *UAS-Alkbh7^o/e^/+*, *act-Gal4/+*. Significance is based on the resulting mixed-effects Cox regression model. NS p>0.05, *p<0.05, **p<0.01, ***p<0.001. The same data for *D. sechellia* are shown in both plots in C. (**D**) Survival curves of wild-type (*Dsec07*), and *kraken* (*Dsec\kraken^RFP^*) and *Alkbh7* (*Dsec\Alkbh7^RFP^*) mutant *D. sechellia* in the plate assay with 30 μl OA. Statistics as in A. (**E**) Long-term survivability of the same genotypes as in D (n=60 per condition) in control conditions (no OA) (left) and food supplemented with 0.9% OA (right). Statistics as in A. Raw data in Figure 5—source data 1. Figure 5—source data 1.Octanoic acid survival of *D. melanogaster* and *D. sechellia* transgenic and mutant flies*.*

Given the expression differences observed in the naturally and experimentally evolved OA-resistant drosophilids, we asked whether artificially modulating gene expression would impact OA tolerance. We first performed ubiquitous RNAi of these genes in *D. melanogaster* (Figure 5—figure supplement 1) and assessed the effects on resistance to OA using a short-term, plate-based assay (Jones, 1998) (see Materials and methods). *kraken^RNAi^* led to significantly diminished OA resistance compared to genetic controls, while *Alkbh7^RNAi^* did not have a discernible effect (Figure 5A). We next generated deletion mutants for these genes in *D. melanogaster* (Figure 5—figure supplement 1). Similar to the RNAi experiments, *kraken* mutants, but not *Alkbh7* mutants, displayed reduced resistance to OA (Figure 5B). All genotypes remained fully viable when no OA was added (Figure 5—figure supplement 2).

**Figure 5—figure supplement 1.**
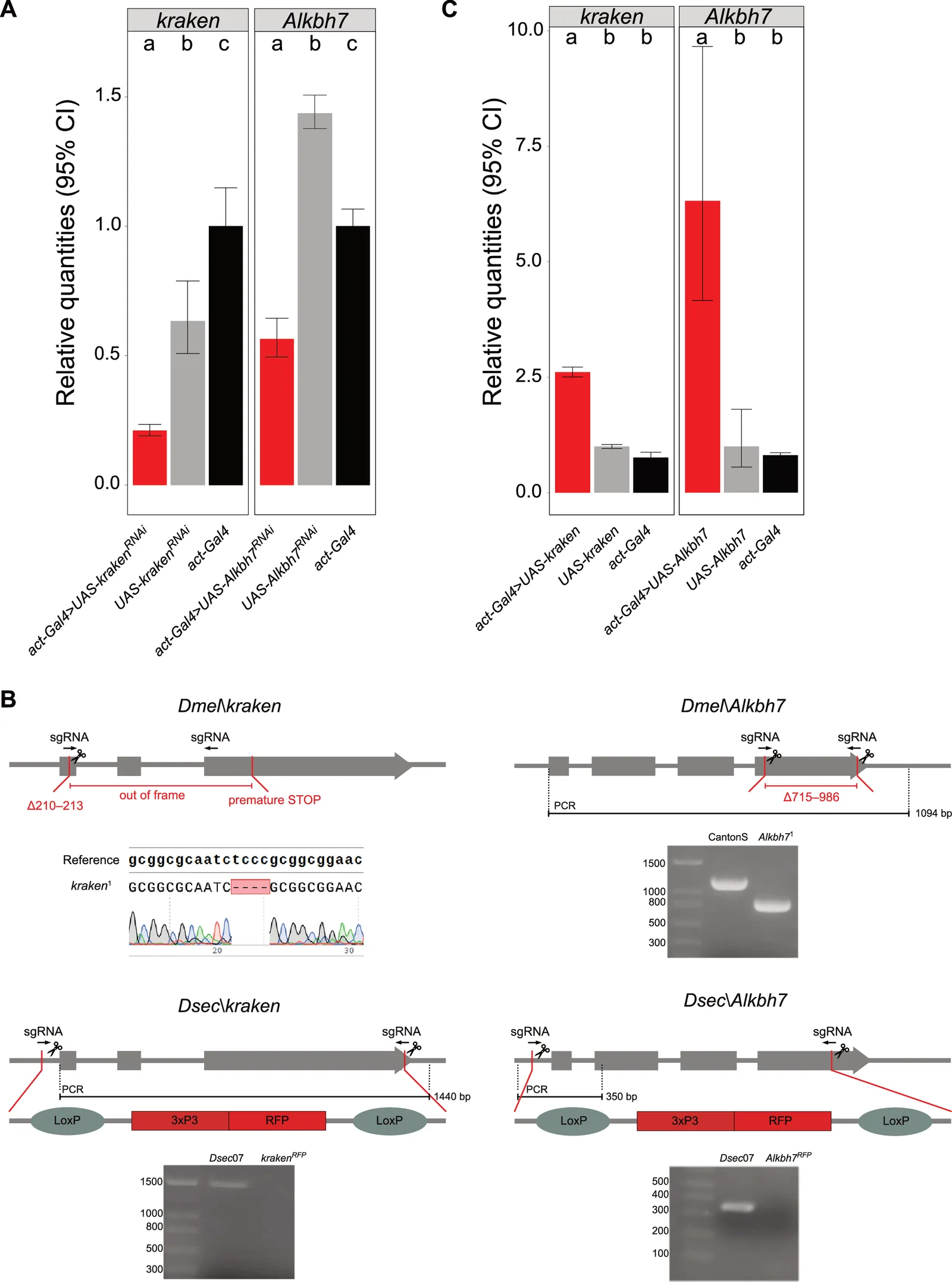
Validation of RNA interference (RNAi) and overexpression lines and generation of mutants for *kraken* and *Alkbh7*. (**A**) Quantitative RT-PCR analysis of RNAi-mediated knockdown of *kraken* and *Alkbh7* in *D. melanogaster*. Genotypes as in Figure 5A. (**B**) Schematics of the CRISPR/Cas9 design to generate *D. melanogaster* and *D. sechellia* mutants for *kraken* and *Alkbh7*. Validation was performed by PCR (Figure 5—figure supplement 1—source data 1) and Sanger sequencing. The *Dse*c\*kraken^RFP^* mutant comprised a 1431 bp deletion (–44 to +1387 nt relative to the ATG), replaced with the 3xP3-RFP cassette. The *Dse*c\*Alkbh7^RFP^* mutant was a 1023 bp deletion (–84 nt to +939 nt relative to the ATG), replaced with the 3xP3-RFP cassette. (**C**) Quantitative RT-PCR analysis of Gal4/UAS-mediated overexpression of *D. melanogaster kraken* and *Alkbh7*. Genotypes as in Figure 5C. Figure 5—figure supplement 1—source data 1.Original agarose gel images of PCR products used to confirm *kraken* and *Alkbh7* mutant genotypes in *D. melanogaster* and *D. sechellia.*

**Figure 5—figure supplement 2.**
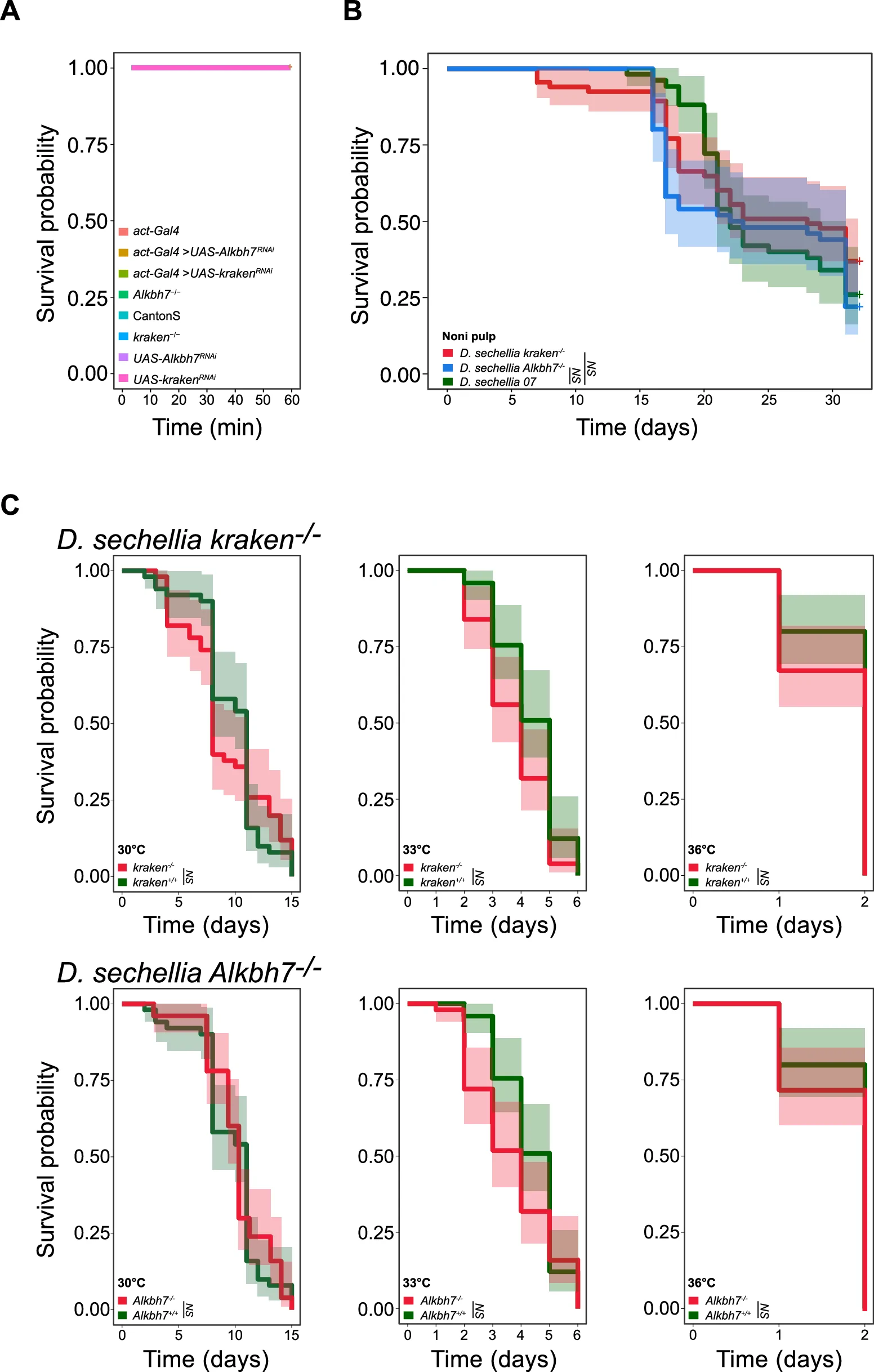
Control survival curves in the absence of octanoic acid (OA). (**A**) Survival curves of adult flies of the indicated genotypes tested in the plate assay without OA. Each replicate consisted of ten 2- to 7-day-old female flies. N replicates ≥5, n flies ≥50. All genotypes display full viability, rendering data points invisible beneath the topmost displayed genotype (*UAS-kraken^RNAi^*). Genotypes as in Figure 5A and B. (**B**) Survival curves of wild-type (*Dsec07*), and *kraken* (*Dsec\kraken^RFP^*) and *Alkbh7* (*Dsec\Alkbh7^RFP^*) mutant *D. sechellia* on homogenized noni pulp. N=50 per strain. Significance is based on the resulting mixed-effects Cox regression model. NS p>0.05. (**C**) Survival curves of *kraken* (*Dsec\kraken^RFP^*, top) and *Alkbh7* (*Dsec\Alkbh7^RFP^*, bottom) mutant *D. sechellia* compared to wild-type (*Dsec07*) flies at 30°C, 33°C, 36°C. N≥50 per strain/condition. Statistics as in **B**.

Loss-of-function analyses might fail to uncover a role for *Alkbh7* in whole animals if there are redundant resistance mechanisms absent from S2R+ cells. We therefore took a complementary approach by overexpressing these genes in *D. melanogaster*. Strikingly, overexpression of *Alkbh7* resulted in survivability of *D. melanogaster* to levels close to those observed for *D. sechellia* under the same experimental conditions (Figure 5C, Figure 5—figure supplement 1), indicating that this gene is sufficient to confer high-level OA resistance. By contrast, *kraken* overexpression did not have any detectable impact on OA resistance (Figure 5C); a similar lack of effect was seen when overexpressing *D. sechellia kraken* (Figure 4—figure supplement 1). The absence of a phenotypic effect upon *kraken* overexpression is surprising, given its elevated expression in *D. sechellia* and in evolved *D. simulans,* but this could reflect either insufficient overexpression levels (Figure 5—figure supplement 1C) and/or genotype-by-genotype interactions that are not recapitulated when manipulating a single locus in a *D. melanogaster* background.

A key question is whether these genes contribute to OA resistance of *D. sechellia*. Implementing CRISPR/Cas9-based genome engineering in this species (Auer et al., 2020; Auer et al., 2021), we generated and validated null mutants in these genes in *D. sechellia* (Figure 5—figure supplement 1). These mutant flies were homozygous viable and fertile, with no obvious morphological or behavioral abnormalities, allowing us to test their resistance to OA in the plate assay. Although we did not detect any significant difference to genetically matched wild-type flies (Figure 5D), this was anticipated given the polygenic nature of OA resistance in *D. sechellia* (Amlou et al., 1997; Amlou et al., 1998; Jones, 1998; Huang and Erezyilmaz, 2015), and the modest or absent phenotypes of equivalent mutants in *D. melanogaster* (Figure 5B). Likewise, rearing *D. sechellia* null mutants on noni pulp (Figure 5—figure supplement 2), we observed no significant differences between mutant and wild-type flies over >30 days, indicating that loss of either gene alone does not compromise *D. sechellia*’s capacity to persist in its natural host fruit.

We reasoned that more subtle differences might be detected by monitoring *D. sechellia* survivability at higher OA concentrations over several days. We therefore exposed flies to food containing 0.9% OA, approximately threefold higher than the ~0.3% OA reported for ripe noni fruit (3.06 g OA/kg fruit) (Pino et al., 2009). Indeed, we observed higher mortality rates of both *kraken* and *Alkbh7* mutants compared to genetically matched wild-type flies over a period of 6 days (Figure 5E). By contrast, both mutants survived equally well as controls in the absence of OA over this time period (Figure 5E). To determine whether the observed OA sensitivity was attributable to a general reduction in fitness, we examined survivorship under several conditions of thermal stress (Figure 5—figure supplement 2): mutant and wild-type flies showed no significant differences across all temperatures tested, indicating that the increased mortality observed upon OA exposure does not result from an overall physiological weakness of the knockout lines. These data provide the first evidence for the contribution of specific genes to OA resistance in *D. sechellia*. However, future reciprocal hemizygosity tests (Stern, 2014) and/or allelic swap studies with *D. simulans* or *D. melanogaster* would be required to assess if and how they contribute to species-specific differences in OA sensitivity (e.g. through structural and/or regulatory differences in the genes).

## Discussion

Identifying specific loci underlying phenotypic evolution is challenging due to the polygenicity of the vast majority of adaptive traits, with recent advocacy of new experimental paradigms to bridge genotype-phenotype relationships (Tautz et al., 2026). Investigating the evolution of chemical toxicity is an attractive model trait because of the relatively easy phenotypic read-out. To gain insight into the genetic basis of susceptibility and resistance to a natural toxin, OA, we have intersected the results of selection over vastly different timescales: weeks in cell lines, years in experimentally evolved laboratory strains, and millennia during speciation.

Unlike insecticides that activate or antagonize broadly expressed neuronal ion channels, the mode of action of OA appears to be mechanistically very different. Beyond insect cell lines (our work and Kaczmarek et al., 2022), OA is also toxic for budding yeast (Mota et al., 2024) and bacteria (Liu et al., 2013), suggesting that this toxin acts via a more fundamental cellular mechanism. Indeed, a genome-wide, knock-out screen in yeast for genes required for tolerance of OA highlighted contributions of several core cellular processes, such as vesicle trafficking, mitochondrial function, or chromatin regulation (Mota et al., 2024), reminiscent of our CRISPR screen results. Consistent with this idea, the results of our experimental evolution and CRISPR screening, as well as earlier mapping efforts in *D. sechellia*/*D. simulans* hybrids (Amlou et al., 1997; Amlou et al., 1998; Jones, 1998; Huang and Erezyilmaz, 2015), revealed multiple genomic regions/genes contributing to the resistance to OA.

Considered separately, the experimental evolution and cell-based CRISPR screens identified many candidates. That there was very limited overlap between the screen results is unsurprising given their very different designs. The loss-of-function, cell-based screen, of course, has several limitations, as it cannot identify genes that are not expressed in the cell line, those essential for cell viability in the absence of OA, nor genes that function in biological processes only pertinent at a tissue/organismal level (e.g. cuticle biogenesis, neuronal signaling). Nevertheless, both lists are likely to contain multiple genes relevant to understanding OA susceptibility and resistance, and hence mode(s) of action of OA. Here, we focused on two resistance gene candidates, *kraken* and *Alkbh7*, which were common hits of both screens. We note that neither of these genes was identified in previous studies of OA resistance (Amlou et al., 1997; Amlou et al., 1998; Jones, 1998; Huang and Erezyilmaz, 2015), which could reflect several factors, such as differences in OA toxicity assay conditions, developmental stages, species, and molecular readouts (DNA or RNA), and the evident multilayered, polygenic nature of this trait (discussed further in Marconcini et al., 2026). However, our recent GWAS of OA resistance in the founder panel of *D. simulans* used to establish the experimental evolution populations identified *bez* and CG13003 as candidate genes (Marconcini et al., 2026), which showed significant CMH signals at generation 25 (but not generation 50) of the Evolve-and-Resequence experiment. (Neither gene could have been recovered in the CRISPR screen because they are not expressed in S2R+ cells; Hu et al., 2017.) The distinction between genetic and adaptive architecture is important in interpreting these results. GWAS identifies genetic variants contributing to phenotypic variation in the founder population, whereas the Evolve-and-Resequence experiment identifies variants whose frequencies changed under selection in our experimental conditions. These approaches, therefore, need not identify the same variants or even the same genes, and the absence of a GWAS signal for an experimentally selected candidate does not exclude a role in adaptation under the selection regime used here.

The (partial) necessity, but insufficiency, of *kraken*, and lack of necessity but sufficiency of *Alkbh7* to confer OA resistance, implies fundamentally different roles of these proteins. The expression of *kraken* in the gut and renal system supports a hypothesis of this protein as a detoxification enzyme, although future work will be necessary to determine whether Kraken has a direct role in OA degradation. The mild loss-of-function phenotype of *kraken* in both *D. melanogaster* and *D. sechellia* likely reflects substantial redundancy of defense mechanisms, including other potential detoxification mechanisms, especially given the plethora of enzymes expressed in the fly gut and Malpighian tubules (Lemaitre and Miguel-Aliaga, 2013; Dow et al., 2022). Indeed, extremely few associations between such enzymes and substrates and/or physiological roles have previously been defined. One exception is the Cytochrome P450 gene *Cyp6g1*, which has a key role in Malpighian tubules in conferring resistance to DDT (Yang et al., 2007). Our data on Kraken, therefore, provide one of the first links between a natural toxic chemical and a putative detoxification enzyme.

The mitochondrial Alkbh7 enzyme likely has a distinct role in OA resistance. Insights from mammals are informative: mice lacking *Alkbh7* are obese, with higher body fat levels, proposed to result from a defect in metabolism of fatty acids (Solberg et al., 2013). Molecularly, one study suggests that Alkbh7’s metabolic role stems from its regulation of RNA editing to control mitochondrial protein translation (Zhang et al., 2021), although it is unclear whether this is its only function. While the *Alkbh7* loss-of-function phenotype is mild in drosophilids, it is striking that artificially elevated expression in *D. melanogaster* is sufficient to produce *D. sechellia*-like levels of OA resistance. This result implies a potent effect of Alkbh7 on the mitochondrial metabolic network to modulate toxin resistance, perhaps through control of fatty acid metabolism. While such ideas can be tested in future studies, we note that defects in lipid metabolism and mitochondrial function have been observed upon exposure to other types of insecticide (Martelli et al., 2020; Martelli et al., 2022). Intriguingly, our implication of Alkbh7 in the niche adaptation of *D. sechellia* has a potential parallel in bats, where this gene displays evidence of positive selection during dietary diversification of different species (Gutiérrez-Guerrero et al., 2020).

An important limitation of the experiments on *kraken* and *Alkbh7* is that the phenotypes produced by a null mutation or overexpression do not necessarily reflect the effect of naturally occurring variation at the same locus. Thus, while our experiments demonstrate that *kraken* and *Alkbh7* can influence OA resistance, they do not establish that their naturally occurring variants have effects of comparable magnitude. Nevertheless, such functional perturbations provide a direct experimental means of testing whether candidate genes can contribute to a complex phenotype, despite the challenges of resolving the effects of individual loci.

Taken together, our work has demonstrated the power of orthogonal selection approaches in the laboratory to understand toxin resistance, which might be relevant to understanding a historic paradigm of this phenomenon (R’Kha et al., 1991), as well as offering clues as to how the natural toxin OA exerts its lethal effects. Moreover, the methodology – notably cell-based CRISPR screening, which can now be extended to both gain-of-function approaches (Xia et al., 2023) and mosquito cell lines (Viswanatha et al., 2021) – is applicable to explore the molecular mechanisms of susceptibility and resistance of insects to the wide range of artificial toxic chemicals used in the environment (Gandara et al., 2024). Such knowledge could have application in the future characterization of novel types of insecticides.

## Materials and methods

### *Drosophila* strains and husbandry

*Drosophila* stocks were maintained on standard wheat flour-yeast-fruit-juice medium under a 12 hr light:12 hr dark cycle at 25°C. Wild-type strains, as well as mutant and transgenic lines used in this study, are listed in Supplementary file 1. Experimental evolution was performed using a subset of published *D. simulans* isogenic strains (Signor et al., 2018; Supplementary file 2).

### Generation of mutant and transgenic flies

#### Loss-of-function mutants

We generated *D. melanogaster* mutants for *kraken* and *Alkbh7* using in vivo CRISPR/Cas9 mutagenesis (Port et al., 2020) by crossing flies expressing Cas9 in the germline (*nanos-Cas9*) to those expressing sgRNAs targeting the desired gene. For *kraken*, we used *P{hsFLP}1,y^1^,w^1118^;P{HD_CFD02326}attP40* as a source of sgRNAs driven by *Act5C-GAL4*; for *Alkbh7*, we used the CG14130 sgRNA line described in Lenče, 2021. Mutants were validated by genomic PCR and sequencing (Figure 5—figure supplement 1).

*D. sechellia* mutant flies were generated by WellGenetics Inc using modified methods of Kondo and Ueda, 2013. For *kraken* (GM16772/LOC6617307), the upstream sequence CTGTAATGCTGGACGCGGAT[TGG] (PAM in square brackets) and the downstream sequence CCTGGTCACCCCGGATCGAG[TGG] were cloned separately into a U6 promoter plasmid. The 3xP3-RFP cassette, which contains a floxed 3xP3-RFP flanked by a 5’ homology arm (999 bp: from –1043 nt to –45 nt relative to the *kraken* ATG) and a 3’ homology arm (1063 bp: +1388 nt to +2450 nt), was cloned into *pUC57-Kan* as a donor template for repair. For *Alkbh7* (GM25300/LOC6605344), the upstream sequences ACTAATAAGAGTCGACCGGG[TGG], AACAGTCAAACAGCTGTTCC[TGG], and downstream sequences GTCCCAGTTTCAGGGTACGC[TGG], ATGGAAATGCGACGTGTCCT[GGG] were cloned separately into a U6 promoter plasmid. The 3xP3-RFP cassette, which contains a floxed 3xP3-RFP, a 5’ homology arm (1037 bp: –1121 nt to –85 nt relative to the *Alkbh7* ATG), and a 3’ homology arm (964 bp: +940 nt to +1903 nt), was cloned into *pUC57-Kan*. DNA plasmids encoding the sgRNAs and the corresponding homology-directed repair template were microinjected into embryos of *Dsec.07 pBAC(nos:Cas9,3P3-YFP)* (Auer et al., 2020). In both cases, a single F1 line expressing the 3xP3-RFP selection marker was obtained, which was validated by genomic PCR and sequencing (Figure 5—figure supplement 1).

*D. melanogaster* and *D. sechellia* mutant flies were backcrossed for six generations to Canton-S and *Dsec*07, respectively.

#### Transgenic flies

To make the *UAS-Dmel\kraken* and *UAS-Dsec\kraken* transgenes, we amplified (using oligonucleotides listed in Supplementary file 6) the regions from the start to the stop codons from *w^1118^* and *Dsec07* genomic DNA and subcloned them into a *pUASTattB* vector (Bischof et al., 2007). Transgenic flies were obtained by phiC31 integrase-mediated transgenesis into attP2 performed by BestGene Inc and verified by sequencing.

### Toxin resistance assays

#### Tube assay

Resistance to OA for the experimental evolution setup was scored adapting a previous protocol (Amlou et al., 1997; Colson, 2004). In brief, flies were lightly anesthetized with CO_2_ and ~50 animals were placed in standard cultured tubes (25 mm diameter × 95 mm height, Milian SA) and provided with 1 ml of a 3% glucose solution on a tissue paper (Kimtech, 7552). 3 μl of pure OA (Sigma-Aldrich, CAS 124-07-2, C2875) were directly pipetted onto the tissue while the flies remained anesthetized. Direct contact with the acid was scrupulously avoided until all flies recovered. The fraction of alive flies was scored after 24 hr. For the experimental evolution experiment, the surviving flies were collected (see below).

#### Plate assay

To measure OA resistance in the genetically manipulated *D. melanogaster* and *D. sechellia* strains, we used a plate assay (Jones, 1998). Compared to the experiments in tubes, this assay offers greater temporal resolution and higher dynamic range (in contrast to the experimental evolution experiment, we did not strive to maintain a certain fraction of surviving flies), as well as greater statistical power from tests of fewer animals. Ten female flies were lightly anesthetized with CO_2_ and placed in a Petri dish (60 mm diameter × 15 mm height) in which 2 μl of pure OA (or 30 μl for *D. sechellia*) were spotted in the center of the underside of the lid. The flies were allowed to recover (typically <5 min), and immobile/dead were scored every 5 min for 1 hr. The survival curves were calculated and plotted using the R package survminer (10.32614/CRAN.package.survminer). The statistical analysis was conducted fitting a mixed-effects Cox regression model as implemented in the R package coxme (Therneau, 2024). Genotype was included as a fixed effect, and replicate dishes were modeled as a random effect.

#### Noni resistance assay

Noni fruit was collected from *M. citrifolia* plants grown in the University of Lausanne greenhouses. Five ripe (pale yellow) fruits were homogenized together, and the pulp puree frozen at –20°C. The noni assay was performed with the same setup as for the tube assay described above, using 2 g of thawed noni puree placed on the bottom of the tube. Noni contains 3.06 g OA/kg fruit (Pino et al., 2009); assuming an equivalent quantity in our fruits, 2 g noni pulp should contain 6.73 µl OA, approximately double the amount used for the experimental evolution between generations 1–25, and two-thirds of the concentration during generations 31–50.

#### Long-term assay

To assess the long-term effects (i.e. beyond 24 hr) of OA or noni pulp, 2-day-old mated female flies were individually housed in vials containing either food supplemented with OA or 2 g of noni puree. OA-supplemented food was prepared by thoroughly mixing OA into Formula 4–24 Instant Drosophila Medium, Blue (Carolina Biological Supply) to a final concentration of 0.9%. Flies were flipped into fresh food every 2 days, and mortality was monitored daily. Statistical analysis was run as for the plate assay.

#### Fecundity and developmental viability

10 mated adult female flies (4–7 days of age) were allowed to lay eggs for 24 hr on agar plates (2% agar, 2.5% sucrose in a 1:3 grape juice:water mix). The total number of eggs on each plate was counted. From each plate, 100 eggs were collected and transferred to standard fly food. Emerging adults were counted and removed daily until no further flies emerged.

#### Lifespan

Two-day-old mated female flies were individually housed in vials containing standard fly food and monitored daily until death. Fresh food was provided every 2–3 days.

### Experimental evolution

#### Selection procedure

To generate the base populations for experimental evolution, we used 93 isogenic lines (Supplementary file 2) of the *D. simulans* panel established by Signor et al., 2018. We first crossed each of the lines together following a round-robin design to ensure equal contribution of each line to the starting genetic pool; five males and five females from each cross were then combined in 10 independent replicates (Figure 1D). These 10 populations were maintained in bottles (57 mm diameter × 103 mm height, Milian SA) of standard fly food at high population size (~1000 individuals per population) to minimize the effect of genetic drift. Each population was subdivided into smaller groups of ~250 individuals per bottle (i.e. four bottles per population) to reduce competition. At each generation, individuals from the four bottles were mixed to prevent bottle-specific genetic drift. The experimental evolution experiment was initiated 10 generations after the creation of these populations.

From each population, 500 individuals (2–7 days of age) were randomly collected and further split into 10 groups of 50 mixed-sex flies (i.e. ~5000 flies total). Each group of 50 flies was then placed under selection with 3 μl of OA for 24 hr (as described above); the survivors were collected and allowed to breed in food vials for 6–8 days, transferring them to fresh vials every 2–3 days. In the next generation, 500 flies per population were collected only from the last seeded vial, to maximize the probability of these flies being parented by the OA-resistant males and females and not offspring generated by females that mated before the selection procedure. These flies were then subjected to the same selection procedure. This process was repeated for 25 generations, and survivability was scored at each generation.

A second round of experimental evolution was performed by increasing the amount of OA to 9 μl between generations 31 and 50; this amount was determined empirically during generations 26–31 to re-establish the survivability close to the initial level (Figure 1G). All successive generations were maintained following the same procedure.

As a control, the 10 original populations were maintained in parallel in the same conditions as previously described (i.e. ~1000 individuals per population subdivided into smaller groups of ~250 individuals per bottle).

#### DNA sequencing and analysis

We pooled ~200 individual flies per population per time point (generations 0, 25, and 50). Genomic DNA (and total RNA for quantitative PCR [qPCR], as described below) extraction was performed using the Quick DNA/RNA Miniprep plus kit (Zymo Research, D7003) following the manufacturer’s instructions. Samples were stored at –80°C.

We performed paired-end whole-genome sequencing on an Illumina NovaSeq 6000 following Nextera DNA flex preparation, at an average coverage of >200×. Read quality was assessed with FastQC (Wood, 2011) and adapters were trimmed with Trimmomatic (Bolger et al., 2014) providing the Nextera adapters sequences. We aligned the trimmed reads to the *D. simulans* reference genome (NCBI GCF_016746395.2) using bwa-mem (Li, 2013), and the results were sorted and compressed using samtools (Danecek et al., 2021). Duplicate reads were then removed with Picard MarkDuplicates (https://broadinstitute.github.io/picard/). Unplaced scaffolds and alignments with mapping quality <20 were removed with samtools. We then combined all the aligned sequences into a samtools pileup file and split this into individual chromosomes. Positions with zero read coverage in any population were excluded from the pileup files prior to downstream analyses, using an awk-based filtering command. Sync files for each chromosome were generated with the mpileup2sync.pl script implemented in Popoolation2 (Kofler et al., 2011) and used as input files for all the Evolve-and-Resequence analyses.

To explore the genetic variation of our samples, we generated vcf files with vcftools 0.1.17 (Danecek et al., 2011) and binary files with plink1.9 (Purcell et al., 2007; Purcell, 2020). We calculated the IBS matrix as implemented in plink1.9 (function -distance ‘ibs’).

#### Evolve-and-Resequence analysis

Allele frequency changes between generation 0 and generation 25 or 50 were investigated via Popoolation2 and Bait-ER (Barata et al., 2023).

We performed a CMH test via the cmh-test.pl script implemented in Popoolation2 comparing base and evolved populations with parameters --min-count 12 and --min-coverage 50. We estimated a plausible significance threshold approximating the top 0.000005% of the simulated p-values based on the effect of genetic drift alone by running genome-wide forward simulations under neutrality in mimicree2 (Vlachos and Kofler, 2018). We used the same genomic information from the original 93 lines as input, with the average real survivability ratios observed throughout the selection experiment as the selection-regime parameter and 500 as the population-size parameter (Figure 2—figure supplement 1).

For Bait-ER, the input sync files were filtered to match --min-count 12 and --min-coverage 50 and scaled to a uniform coverage of 100 as in Vlachos et al., 2019, to avoid numerical problems due to high coverage. We ran the algorithm on each chromosome separately. Bait-ER models the effect of genetic drift directly using estimates of the effective population size (N_e_). We calculated N_e_ estimates for each chromosome in each replicate population with the R package poolSeq (Taus et al., 2017) and used the median value of each chromosome as ‘Population_size’ parameter (Supplementary file 3). Other parameters included ‘Sampling correction’=0 (i.e., binomial) and ‘Prior parameters’=0.001 (shape), 0.001 (rate). Data points were considered significant when the absolute natural logarithm of the Bayes factor exceeded the conservative threshold of log(0.99/0.01) as in Barata et al., 2023.

The linkage disequilibrium decay in the 10 evolved populations was analyzed across the whole genome using PopLDdecay (Zhang et al., 2019). Linkage disequilibrium maps of focal regions were generated with LDBlockShow (Dong et al., 2021) using the Solid Spine method based on D’ statistics originally introduced in Haploview (Barrett et al., 2005).

For the genome-wide screen, all the genes within selection blocks were considered potential candidates. Based on the results on linkage disequilibrium decay, a block was arbitrarily defined as a genomic interval where at least two significant SNPs were found at a distance of less than 50 kb. Consecutive SNPs respecting this definition were grouped into larger blocks and visualized as circular plots using the R package circlize (Gu et al., 2014). The genes overlapping such blocks were extracted using BEDTools intersect (Quinlan and Hall, 2010). Orthologous genes in *D. melanogaster* were retrieved using a combination of the OMA browser (Altenhoff et al., 2021), DAVID (Huang et al., 2009; Sherman et al., 2022), BLAST (Camacho et al., 2009), NCBI (Sayers et al., 2025), and FlyBase (Öztürk-Çolak et al., 2024), and subsequent manual curation.

### Genome-wide CRISPR/Cas9 screen in *D. melanogaster* S2R+ cells

#### Library design, construction, and transfection

Design, synthesis, and cloning of the genome-wide sgRNA library are previously described (Viswanatha et al., 2025). To determine sub-lethal doses of OA appropriate for screening, the effect of a gradient of OA supplementation into media on attP+ Cas9+ cells was determined using CellTiter-Glo (Promega) following the manufacturer’s protocol; luminescence measurements were made using a SpectraMax Paradigm (Molecular Devices).

For the screen, attP+ Cas9+ *D. melanogaster* S2R+ cells (RRID:CVCL_UD30) were transfected using Effectene and a 1:1 molar ratio of sgRNA library and pIntAC, as described (Viswanatha et al., 2025). Briefly, transfected cells were distributed in 5 ml aliquots across ten 100 mm culture dishes and incubated overnight; then, 5 ml of fresh media was added to each plate. After 4 days, cells were subjected to puromycin selection for 12 days, subculturing every 4  days, to establish a stable sgRNA-expressing cell library. For the selection assay, 2.5 × 10^7^ cells were plated onto four 15 cm diameter cell culture dishes to ensure sufficient sgRNA coverage, with each sgRNA represented approximately 1000 times (Viswanatha et al., 2025).

#### OA selection assay

Library-containing cells were left untreated or treated with either 0.075 μg/μl or 0.05 μg/μl OA (Sigma-Aldrich, CAS 124-07-2, C2875). Each replicate consisted of four 15 cm diameter cell culture dishes seeded with cells, achieving ~1000× coverage. Two replicate sets were included for each of the two OA treatment conditions and one replicate for the untreated control. During the selection period, cells were split one to two times per week to avoid overgrowth. An aliquot of cells from each replicate was collected and stored for genomic prep following each split and at the end of the assay. The final samples were collected after 26 days of OA or control treatment.

#### Genomic DNA extraction and sequencing

Genomic DNA was extracted from samples collected during the screen and at the final endpoint using a Zymo Quick-gDNA Miniprep kit (Zymo Research, D3025). DNA fragments containing the sgRNA sequences were amplified by PCR using what we calculated to be at least 1000 genomes per sgRNA as templates for each sample. PCR fragments were in-line barcoded using a previously described approach (Viswanatha et al., 2018; Viswanatha et al., 2019; Viswanatha et al., 2025). Next-generation sequencing (Illumina NextSeq) was performed at the Biopolymers Facility at Harvard Medical School (RRID:SCR_007175). FASTQ sequence files were demultiplexed using in-line barcodes as sequence tags, as described previously (Viswanatha et al., 2018; Viswanatha et al., 2025). All further processing was done using MAGeCK (v.0.5.4 and 0.5.9.4) (Li et al., 2014) to generate readcount files and perform robust rank aggregation (RRA) analysis and maximum likelihood estimation directly on the generated readcount files using default parameters. Plasmid readcounts were used to normalize RRA values.

After removing essential genes at a 2% FDR (2517 genes; Figure 3—source data 1), we considered as candidates the top 5% genes overlapping across the two conditions that were over-represented or under-represented in the OA-exposed cell populations compared to the control cells (i.e. representing ‘susceptibility’ and ‘resistance’ genes, respectively). The gene set enrichment analysis was performed with PANGEA 1.1 Beta using the *Drosophila* GO subsets (GO slim) and a p-value cutoff of 0.05 (Hu et al., 2023).

### qPCR analysis

Reverse transcription of total RNA (extracted as described above) was performed with the PrimeScript first-strand cDNA synthesis kit (Takara, 6110A) following the manufacturer’s instructions. Samples were stored at –80°C.

qPCR experiments were conducted on a QuantStudio 6 Flex Real-Time PCR System using the PowerUp SYBR Green Master Mix following the manufacturer’s instructions. Each sample consisted of at least three biological replicates. We performed three technical replicates per reaction and removed replicates where the standard deviation was equal to or greater than 0.5. We determined target-specific amplification efficiencies for each gene (Supplementary file 4, Supplementary file 6). Relative quantification and statistical analysis were performed with the software qbase+ v3.4 (Hellemans et al., 2007) using calibrated normalized relative quantities. Normalization was performed on the geometric mean of the relative quantities for the three most commonly used reference targets: *Act5C*, *αTub84B*, and *RpL32* (Lü et al., 2018). We assessed reference target stability by the geNorm expression stability value (M) and the coefficient of variation of the normalized reference gene relative quantities (CV) (Supplementary file 5; Vandesompele et al., 2002; Hellemans et al., 2007).

### Population genetics analysis

Publicly available WGS data for 41 *D. sechellia* and 20 *D. simulans* genomes were downloaded from the Sequence Read Archive (PRJNA215932 and PRJNA395473) (Rogers et al., 2014; Schrider et al., 2018) with fasterq-dump (Leinonen et al., 2010). FASTQ files were processed and aligned as described in the ‘Experimental evolution’ section. Aligned files were phased with samtools phase (Danecek et al., 2021), and the genes of interest were extracted with pilon (Walker et al., 2014) to find variation among populations. Coding sequences were aligned based on amino acid information using translatorX (Abascal et al., 2010). Gene sequences were aligned using Clustal Omega (Madeira et al., 2024). McDonald-Kreitman test, Tajima’s D, and Fu and Li’s statistics were calculated in DNAsp 6 (Rozas et al., 2017). FUBAR analyses were performed in HyPhy 2.5 (Kosakovsky Pond et al., 2020). The consensus sequences of *D. simulans* and *D. sechellia* were computed by SnapGene (https://www.snapgene.com) ‘export consensus’ with a >50% threshold. The sequence alignment and secondary structures were depicted using ESPript 3 (Robert and Gouet, 2014).

### RNA FISH

To detect *kraken* mRNA in situ, we used the RNAscope Multiplex Fluorescence Detection Reagents v2 (Advanced Cell Diagnostics, 323110) and RNAscope H_2_O_2_ and Protease Reagents (Advanced Cell Diagnostics, 322381). Surfaces and equipment were cleaned with RNAzap (Invitrogen, AM9780) prior to dissection. Intestinal tissues were dissected from *D. melanogaster* (Canton S) and *D. sechellia* (*D. sechellia* 07) females in RNAlater (Sigma-Aldrich, R0901) diluted 1:50 in DEPC-treated water and briefly rinsed in 1× PBS (prepared in ultrapure water) (Thermo Fisher Scientific, 70011044). Dissected tissues were mounted on poly-L-lysine-coated slides in 1× PBS and fixed immediately in cold 4% formaldehyde (Sigma-Aldrich, F8775) for 30 min. The fixative was removed, and tissues were incubated with a few drops of hydrogen peroxide for 10 min at room temperature (RT). Tissues were then washed 3×5 min in wash buffer (Advanced Cell Diagnostics, 320058) preheated to 40°C. For tissue dehydration, slides were incubated with 200 μl of ethanol solutions in the following order: 50% ethanol for 5 min at RT, 70% ethanol for 5 min at RT, and 100% ethanol for 5 min at RT (repeated twice). Slides were air-dried for 5 min, and samples incubated with a few drops of Protease III for 30 min at RT.

To hybridize probes, tissues were washed in 1× PBS for 3×5 min. 50 μl of C1 (*D. melanogaster-kraken-RA* [NM_057917], Advanced Cell Diagnostics, 1850601-C1); and 1 μl of C2 (*D. melanogaster-Rpl32* [NM_079843.4], Advanced Cell Diagnostics, 533451-C2) RNAscope FISH probes were preheated to 40°C for 10 min, cooled at RT for 5 min, applied to tissues, and the slides were placed in a humidified chamber for hybridization at 40°C for 2 hr. Slides were hybridized with AMP reagents in sequential steps. A few drops of AMP1, warmed to RT, were applied to slides, which were incubated at 40°C for 30 min, followed by washes in wash buffer 3×5 min. Next, a few drops of AMP2, warmed to RT, were applied to slides and incubated at 40°C for 30 min, followed by washes in wash buffer 3×5 min. Finally, a few drops of AMP3, warmed to RT, were applied to slides and incubated at 40°C for 15 min, followed by washes in wash buffer 3×5 min.

A few drops of HRP-conjugated probe were applied for C1, and slides were incubated at 40°C for 15 min. After rinsing in wash buffer 3×5 min, Opal dyes 520 nm (Akoya Biosciences, OP-001001) and/or 570 nm (Akoya Biosciences, OP-001003) (1:1000 in TSA multiplex buffer, Advanced Cell Diagnostics, 322809) were applied to slides and incubated at 40°C for 30 min. Slides were then washed and treated with HRP blocker (a few drops per slide) for 15 min at 40°C and washed in wash buffer for 3×5 min. Tissues were counterstained with DAPI for 5 min at RT and washed once in the wash buffer before mounting in Vectashield (Vector Laboratories, H1000).

A Leica STELLARIS-DIVE confocal microscope was used to generate all confocal images, using a water-immersive 40× objective or an oil-immersive 63× objective. The images were acquired using both HyDs and PMTs tailored for the fluorophores of each sample.

### Statistics and reproducibility

Data were analyzed and plotted in R 4.0.3 (R Core Team 2021).

## Data Availability

Source data is provided in the supplementary data files and at Dryad (https://doi.org/10.5061/dryad.jwstqjqrt). The following dataset was generated: MarconciniM BentonR 2026Experimental evolution data of *Drosophila simulans* selected with octanoic acidDryad Digital Repository10.5061/dryad.jwstqjqrt

## Additional files

Supplementary file 1. Drosophilid strains.

Supplementary file 2. *D. simulans* lines used to generate the base populations.

Supplementary file 3. Effective population size estimates from the experimentally evolved populations.

Supplementary file 4. Quantitative PCR (qPCR) primer efficiency.

Supplementary file 5. geNorm analysis of reference target stability.

Supplementary file 6. Oligonucleotides.

MDAR checklist
