# Supplementary material for: Intersecting experimental evolution and CRISPR screens to identify novel toxin resistance loci
Source: eLife. 2026 Sep 11;15:RP111773. doi: 10.7554/eLife.111773 (PMC13568702; doi:10.7554/eLife.111773)
Supplement: Supplementary file 1 [file elife-111773-fig5-figsupp1-data1.pdf]

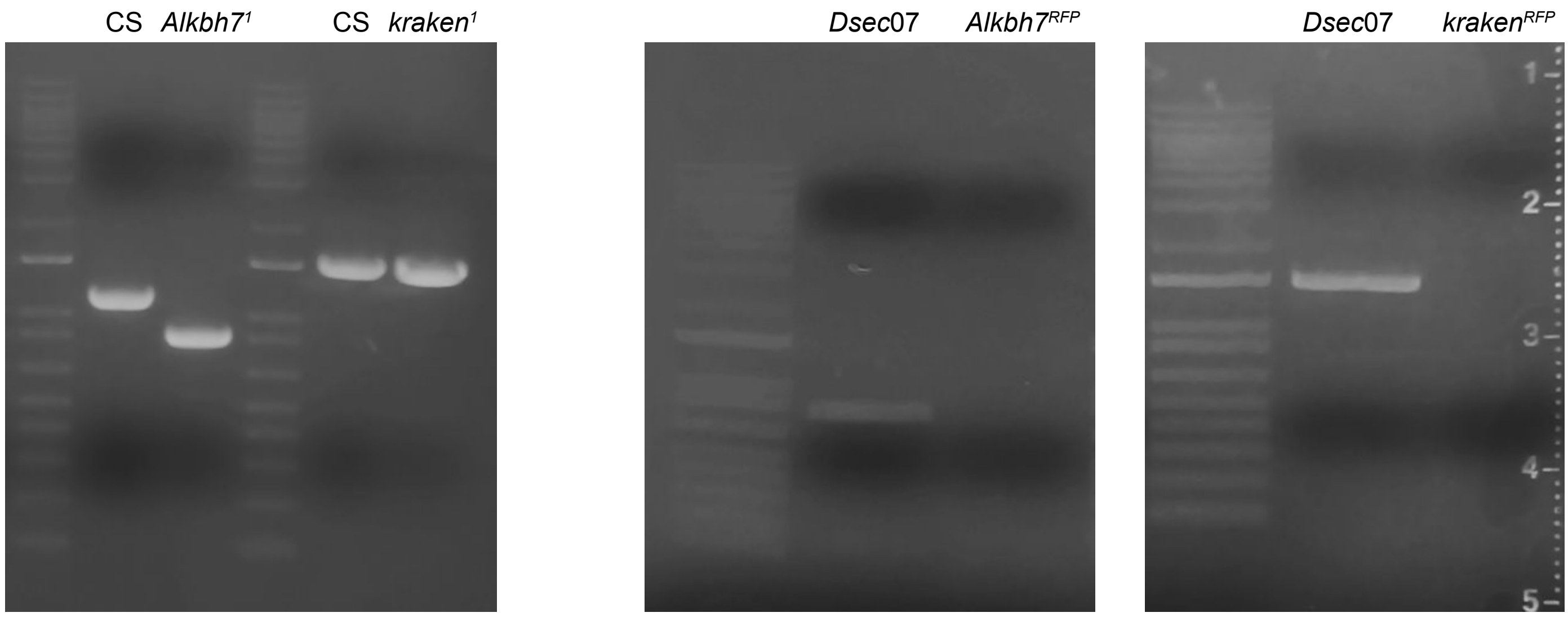
CS
Alkbh7^{1}
CS
kraken^{1}
*Dsec*07
Alkbh7^{RFP}
*Dsec*07
kraken^{RFP}
1
2
3
4
5
