# Supplementary material for: Intersecting experimental evolution and CRISPR screens to identify novel toxin resistance loci
Source: eLife. 2026 Sep 11;15:RP111773. doi: 10.7554/eLife.111773 (PMC13568702; doi:10.7554/eLife.111773)
Supplement: Peer review history

# Editor report: eLife Assessment

**DOI:** 10.7554/eLife.111773.3.sa0

**Authors:** Virginie Courtier-Orgogozo

**Affiliations:** CNRS - Universite Paris Cite France

**Keywords:** Important, Solid

This **important** study presents an innovative combination of experimental evolution and CRISPR screening to study the genetic basis of a polygenic trait, resistance against octanoic acid in Drosophila. The evidence supporting the authors claims is **solid**. The work will be of interest to the Drosophila community and researchers interested in functional testing of polygenic traits.

# Referee report: Reviewer #2 (Public review)

**DOI:** 10.7554/eLife.111773.3.sa1

Summary:

The authors studied the resistance against octanoic acid, a compound of the noni fruit in D. simulans, using experimental evolution and resistance/susceptibility in *D. melanogaster* cells. They identified novel candidate genes and performed functional tests.

Strengths:

The idea of using experimental evolution of a non-resistant species to develop resistance is interesting and the idea of narrowing down a large list of candidate loci by CRISPR based gene knockout in cell culture is innovative. The reviewer also liked the (easy) follow up experiments to validate the results.

Comments on revised version.

Weaknesses:

- The experiments to validate the effect of candidate genes did not match the experimental evolution conditions.

This point has been confirmed by the authors.

- The statistical analysis suffers from some problems and insufficient description of the analyses performed.

Has not been addressed in their response.

- Although D. simulans GWAS data are available, the authors did not make an attempt to estimate the effect of selected variants in the candidate genes in the GWAS data set.

This has now been included in the discussion. I would recommend that they make the distinction between genetic and adaptive architecture, as this matches their verbal description.

- The reviewer would have liked to see more connection between the experimental evolution and GWAS data. As some D. simulans genotypes have similar resistance as D. sechellia, it would have been interesting to test whether this genotype contributed to the observed resistance.

The reviewer is happy with the response.

- At several places the authors discuss the challenge of studying a polygenic trait, but at the same time they claim to have detected and validated candidate genes. It would be helpful if the authors could discuss why they consider that their assays could really detect the contribution of single loci to the polygenic trait. In particular, when GWAS did not detect their candidate genes.

The reviewer is not satisfied with the arm waving explanation of the authors. The important question is how much of the phenotypic variation is explained by the two candidate genes? The reviewer is inclined that based on the weak selection response, the variation is too little to be detected experimentally. Nevertheless, the overexpression of alkbh7 alone was sufficient to generate resistance levels similar to the ones in *D. melanogaster*. Hence, it is not adequate to speak of small effects. This discrepancy requires more discussion.

- It is not clear to the reviewer why the authors did not pay more attention to the highly significant peaks emerging from the experimental evolution study. Their functional validation would have been biologically more plausible.

This point remains valid, in particular in the light of the discrepancy between the very limited selection response of alkbh7 and its large phenotypic effect after overexpression.

Impact:

- Given the obvious challenges of functional testing of polygenic traits and the clear limitations of the interpretation of the results, the study will be helpful for future studies aiming to characterize polygenic traits. Unfortunately, the results are just another piece of controversial results regarding resistance against octanoic acid-a trait that is rather easy to evaluate.

The reviewer did not find the reply satisfactory.

# Author comment: Author response

**DOI:** 10.7554/eLife.111773.3.sa2

**Authors:** Michele Marconcini, Steeve Cruchet, Srishti Goswami, Raghuvir Viswanatha, Matthew Butnaru, Joydeep De, Camilla Roselli, Dafni Hadjieconomou, Norbert Perrimon, Stephanie E Mohr, Richard Benton

**Affiliations:** University of Lausanne Lausanne Switzerland; University of Lausanne Lausanne Switzerland; Harvard Medical School Boston United States; Harvard Medical School Boston United States; Harvard Medical School Boston United States; Institut du Cerveau Paris France; Institut du Cerveau Paris France; Institut du Cerveau Paris France; Harvard University Boston United States; Harvard Medical School Boston United States; University of Lausanne Lausanne Switzerland

The following is the authors’ response to the original reviews.

**Public Reviews:**

**Reviewer #1 (Public review):**

Marconcini et al. report results of an ambitious study on the genetic mechanisms that contribute to resistance of Drosophila flies to the toxin octanoic acid (OA). This study was motivated by two observations: first, Drosophila sechellia, a close relative of *D. melanogaster*, has evolved specialized feeding on fruits of Morinda citrifolia, which contain high concentrations of OA and second, that artificial selection on Drosophila simulans, a sister species of *D. melanogaster*, can generate higher resistance to OA. Previous studies had performed genetic mapping studies between D. simulans and D. sechellia that implicated certain genomic regions in resistance to OA and, in particular, implicated several Osiris gene paralogs as contributing to resistance, though the molecular mechanisms of resistance remain unclear. In this study, Marconcini et al. performed two major experiments. First, they performed evolution-and-resequence on Drosophila simulans populations exposed to OA for 50 generations and identified candidate regions with excessive shifts in allele frequencies as candidate regions containing OA resistance genes in D. simulans. Second, they performed a CRISPR knock-out screen in a *D. melanogaster* cell line to identify genes that contribute to OA resistance and susceptibility.

Evolve-and-resequence yielded many candidate genomic regions with extreme allele frequency shifts, which may be regions containing OA resistance genes, or linked genes, or regions that happen to show a strong shift in all replicate populations by chance. As the authors note, detecting significant shifts in allele frequencies is a challenging problem, and the authors use two measures of allele frequency shifts (the Cochran-Mantel-Haenszel method and Bait-ER) and perform simulations under neutrality to estimate a reasonable significance threshold. I am not entirely convinced by this method of estimating significance levels, because the simulations involve assumptions that may not be met by the real populations. I would think that a permutation test would provide an assumption-free method of estimating significance levels. I have tried to think whether there is something about the design of these experiments that would preclude the use of permutation tests (which are used widely for genome-wide studies, such as QTL), but I can't think of one. Perhaps the authors are aware of a reason permutation tests would be invalid here, and if so, they should state this reason.

Significance thresholds have been estimated using a variety of approaches in the literature, including false discovery rate (FDR) control, simulations under neutral drift with predefined cut-offs, arbitrary significance thresholds, haplotype-based analyses, permutation tests, amongst other methods. Our choice was guided by a review (doi:10.1186/s13059-019-1770-8), which compared the performance of several of these approaches and found that the relatively simple assumptions underlying the Cochran–Mantel–Haenszel (CMH) test often performed as well as, or better than, more complex alternatives. As with most significance thresholds used in genome-wide analyses, the CMH threshold applied here is ultimately based on a degree of arbitrariness, although we aimed to be as stringent as possible. As a complementary method, we used Bait-ER; here the threshold used followed the recommendations provided in the original publication describing this method (doi:10.1111/jeb.14134).

One reason that permutation-based approaches may be less widely adopted than theoretical null models is the extensive linkage disequilibrium among SNPs, which results in large blocks of correlated variants that cannot be considered independent observations (and therefore not shuffled). In principle, permutations could be performed at the haplotype-block level; however, defining haplotype blocks itself requires selecting thresholds or criteria that are often user defined or based on significance cut-offs. Although several tools are available for this purpose, our experience has been that the resulting block definitions remain sensitive to these choices and therefore introduce a comparable degree of arbitrariness. In reality, there is not yet a standard method in the field that has emerged as the “best” one.

There is overlap between regions detected by the two methods, but the methods disagree for many regions. The authors state that a "majority of prominent peaks were found by both methods," but I am unclear on what "prominent" means here. It would be more helpful to be more quantitative about the extent of overlap.

To quantify the agreement between the two methods, we calculated the overlap in genomic coverage (base pairs) between CMH and Baiter candidate regions. For G25, the two methods shared 1.90 Mb of candidate regions. The overlap encompassed 65.3% of the genomic span identified by CMH and 64.1% of the span identified by Bait-ER. For G50, the methods shared 4.77 Mb of candidate regions. The overlap encompassed 63.6% of the CMH candidate span and 99.9% of the Bait-ER candidate span. We have now replaced the admittedly qualitative statement the reviewer highlighted ("majority of prominent peaks were found by both methods”) with this information.

The authors hypothesized that the response would be at similar genomic loci in all populations (line 222). It seems at least possible that epistatic interactions would lead to different combinations of alleles evolving in each population. I wonder if it would be possible to test whether there is heterogeneity in the responses across the replicate populations.

We agree that epistatic interactions could lead to different allelic combinations being favored in different replicate populations. However, both BaitER and the CMH test are designed to detect parallel evolutionary responses across replicates, which was the focus of our study. One approach for testing population-specific responses is the LRT-2 test (doi:10.1534/genetics.118.301824). We did not pursue this analysis because it would likely generate many additional candidate loci, making interpretation more challenging, while providing limited additional insight into the repeatable genomic responses that were the primary focus of this work.

The evolve-and-resequence method yielded many possible regions contributing to OA resistance in D. simulans, but perhaps too many regions to test directly or even to build sensible hypotheses about the genes involved. Thus, the authors performed a second experiment to try to narrow down the list of possible candidate genes. They performed a CRISPR knockout screen in a *D. melanogaster* cell line for genes that contribute to resistance or susceptibility to OA. The authors identify several limitations of this experiment, but they nonetheless identified several genes where knockouts contribute to OA susceptibility or resistance. Intersecting top hits with regions that experienced selection identified two "resistance" genes: kraken and Alkbh7. The selection hit at kraken is quite compelling, whereas the evidence at Alkbh7 is less strong because only two SNPs were marginally significant. Further functional assays, including gene knockouts in *D. melanogaster* and D. sechellia, provide some support for the claim that both of these genes can contribute to resistance to OA in flies.

Beyond the few issues raised above, I do not have significant questions about methodology or the results. I do think, however, that the authors should be more conservative about the implications and significance of their results. For example, on line 139, the authors claim that this intersection approach provides a "powerful paradigm to investigate ecotoxicology." I am not sure I agree that the identification of two genes that may contribute to OA resistance, after a seemingly heroic selection experiment and CRISPR screen, suggests that this method is all that powerful. It seems that most of the genes that contribute to the selection response remain unidentified.

We agree with the reviewer and have modified the sentence accordingly. While we believe that integrating the approaches discussed in this paper can provide valuable insights into the genetic basis of ecotoxicological traits, these approaches are not a panacea for traits with highly complex genetic architectures, such as OA resistance. Nevertheless, the identification of two candidate genes with some evidence of contributing to the trait represents a meaningful advance toward understanding its underlying genetic basis.

Finally, given that one motivation of this project was to identify genes that contribute to evolved resistance to OA, I am surprised that the authors did not generate CRISPR alleles of kraken and Alkbh7 in D. simulans and then use these together with the existing alleles in D. sechellia to perform reciprocal hemizygosity tests to determine if these two genes actually contribute to evolved resistance in D. sechellia. This test is simpler to perform and may be more sensitive than the allelic replacement that the authors propose (lines 446-449).

While generating null alleles for the candidate genes in *D. simulans* is beyond the scope of this revision, we note that we did attempt reciprocal hemizygosity tests using the mutants available in *D. melanogaster*. However, the resulting hybrids were recovered in low numbers, and these animals were rather weak, making them unsuitable for the severe OA exposure conditions employed in our assays. We agree that, where feasible, future studies should incorporate reciprocal hemizygosity tests, as they represent a powerful approach for validating the contribution of candidate genes to the trait of interest. We have revised the final sentence of the Results section accordingly.

**Reviewer #2 (Public review):**

Summary:

The authors studied the resistance against octanoic acid, a compound of the noni fruit, in D. simulans, using experimental evolution and resistance/susceptibility in *D. melanogaster* cells. They identified novel candidate genes and performed functional tests.

Strengths:

The idea of using experimental evolution of a non-resistant species to develop resistance is interesting, and the idea of narrowing down a large list of candidate loci by CRISPR-based gene knockout in cell culture is innovative. The reviewer also liked the (easy) follow-up experiments to validate the results.

Weaknesses:

The reviewer is not convinced of the conceptual idea behind their approach: the intersection of the two approaches implicitly assumes that null alleles (or at least compromised alleles) should be selected during experimental evolution. The reviewer considers this unlikely, and the authors made no attempt to test this implicit hypothesis in their data.

We respectfully disagree with the reviewer’s interpretation of the conceptual idea behind our approach. Our strategy did not assume that experimental evolution selects for null alleles, but rather for any type of variant that could contribute to the trait being selected for (i.e., increases in OA resistance), pointing to candidate genes contributing to the trait. Like many evolve-and-resequence experiments, this approach identified hundreds of candidate genes. This is why we took an orthogonal, genome-wide CRISPR screening approach, where loss-of-function mutations could lead to increases or decreases in OA tolerance of cultured cells. However, we stress that the naturally selected alleles – which could be gain or loss of function – may have much subtler phenotypic effects than the null alleles used for functional validation.

Along the same lines, it is not clear how to reconcile an upregulation of candidate genes in resistant flies with the knockout experiments.

We respectfully disagree that these findings are difficult to reconcile. The observed upregulation of the candidate genes in the selected, resistant *D. simulans* is consistent with a role in promoting OA resistance, while the knockout experiments (whether in cultured cells or in whole animals) demonstrate that loss of gene function reduces resistance. These observations are complementary: increased expression is associated with enhanced resistance, whereas complete loss of function impairs it. The knockout experiments of *kraken* and *Alkbh7* were intended as functional validation of gene involvement and do not imply that the alleles selected during experimental evolution are loss-of-function alleles (we rather hypothesize that the selected alleles are gain-of-function through some, as yet undetermined, mechanism).

The experiments to validate the effect of candidate genes did not match the experimental evolution conditions.

This is correct. As our results suggest that the phenotype is shaped by multiple genes, such that the effect of any individual gene is likely modest compared to their combined contribution. Consequently, detecting and validating the effect of a single gene requires more stringent OA conditions than those needed to observe the overall phenotypic response over the course of several generations. In addition, the shorter-term plate assay was more practical for higher temporal resolution of the analysis of mortality in the presence of OA.

The statistical analysis suffers from some problems and an insufficient description of the analyses performed.

Although D. simulans GWAS data are available, the authors did not make an attempt to estimate the effect of selected variants in the candidate genes in the GWAS data set.

We agree that comparing the experimental evolution and GWAS results (from our previous work, doi:10.1093/g3journal/jkag032) is of considerable interest. We have now expanded the Discussion to explicitly discuss the relationship between the two datasets. Overall, the overlap between the approaches was limited, although two GWAS candidate genes, *bez* and CG13003, fall within genomic regions exhibiting significant CMH signals at generation 25 (but not generation 50) of the evolve-and-resequence experiment. (We note that these genes could not have been identified in our CRISPR screen, as they are not expressed in S2R+ cells). More broadly, differences between the GWAS and evolve-and-resequence results likely reflect the distinct evolutionary processes captured by each approach: GWAS maps standing phenotypic variation among isofemale lines, whereas experimental evolution tracks allele frequency changes under sustained selection. Understanding why some signals are shared whereas others are not – whether due to effect size, genetic background, epistasis, pleiotropic costs, or the contribution of initially rare variants – remain important open questions.

The reviewer would have liked to see more connection between the experimental evolution and the GWAS data. As some D. simulans genotypes have similar resistance to D. sechellia, it would have been interesting to test whether this genotype contributed to the observed resistance.

While *D. simulans* genotypes displayed a range of OA resistance levels, none approached *D. sechellia* levels of resistance (see Figure 2e from our previous work, doi:10.1093/g3journal/jkag032) (The reviewer might have conflated the data from our GWAS of *D. melanogaster* strain, shown in Figure 2b of that paper, where some lines of that species exhibit comparable resistance to *D. sechellia* under the conditions of that assay). Regardless, our experimental evolution data do not provide sufficient resolution to identify the specific favorable alleles underlying the response to selection. Instead, we detect genomic regions containing many linked variants whose frequencies change under selection. Consequently, a direct comparison between evolved genotypes and GWAS-associated genotypes is currently difficult. We note, however, that expression of the *D. sechellia kraken* allele in *D. melanogaster* did not produce a significant effect on resistance, suggesting that even the most promising candidate alleles might not have strong effects in isolation.

At several places, the authors discuss the challenge of studying a polygenic trait, but at the same time, they claim to have detected and validated candidate genes. It would be helpful if the authors could discuss why they consider that their assays could really detect the contribution of single loci to the polygenic trait. In particular, when GWAS did not detect their candidate genes.

Our results do not imply that *kraken* and *Alkbh7* are major-effect loci or that they explain a substantial proportion of the phenotypic variation. Rather, our data indicate that these genes make measurable contributions to OA resistance, consistent with the expectation that complex traits are influenced by many loci of individually modest effect. The absence of these genes among the top GWAS candidates does not preclude their involvement, as GWAS and experimental evolution interrogate different aspects of the genetic architecture and differ in their power to detect loci of varying effect sizes and allele frequencies.

It is not clear to the reviewer why the authors did not pay more attention to the highly significant peaks emerging from the experimental evolution study. Their functional validation would have been biologically more plausible.

We agree that the significant peaks identified in the evolve-andre sequence experiment represent promising targets for future investigation. However, these peaks typically span large genomic regions containing tens to hundreds of genes, making it difficult to prioritize individual candidates based on the experimental evolution data alone. In this work, we focused our functional validation on genes independently supported by the CRISPR screen, which provided gene-level resolution. We fully acknowledge that additional causal genes are likely to reside within the selected regions and remain to be functionally characterized.

Impact:

Given the obvious challenges of functional testing of polygenic traits and the clear limitations of the interpretation of the results, the study will be helpful for future studies aiming to characterize polygenic traits. Unfortunately, the results are just another piece of controversial results regarding resistance against octanoic acid, a trait that is rather easy to evaluate.

The reviewer appears to imply that a trait being straightforward to phenotype necessarily implies that its genetic basis should also be straightforward to resolve. Many classic complex traits, such as human height, are simple to measure yet have an extraordinarily complex, highly polygenic genetic architecture. We believe that OA resistance represents a similar challenge: while the phenotype is readily assayed, differences in assay conditions, genetic backgrounds, and the contribution of many loci of individually modest effect make its genetic basis difficult to dissect. We have strived to be cautious in our conclusions, in particular the evolutionary interpretations; nevertheless, to our knowledge, this is the first study to provide functional evidence supporting the contribution of specific genes to OA resistance in *D. sechellia*, combining both loss-of-function phenotypes and expression data. As emphasized by the title of our manuscript, we view the principal contribution of this work as demonstrating how complementary experimental approaches (both of which are fairly novel for study of toxin susceptibility/resistance genetics) can be integrated to prioritize and functionally evaluate candidate genes underlying complex adaptive traits.

**Recommendations for the authors:**

**Reviewing Editor Comments:**

Both reviewers propose to include additional statistical and quantitative measures to strengthen the results. Furthermore, certain parts of the manuscript could be rephrased to make sure that the readers can understand more clearly the implications and significance of the results. To test whether the genes identified in the present manuscript (as contributing to octanoic acid resistance) are also involved in the evolution of the resistance, reciprocal hemizygosity tests using CRISPR alleles of kraken and Alkbh7 in D. simulans would be a plus, but such experiments are not required because the main focus of the paper is on the genetic basis of octanoic acid resistance, and not on evolution.

We thank the Reviewing Editor for these constructive comments. We have revised the manuscript to clarify the interpretation and significance of our findings and have addressed the reviewers’ comments throughout. Regarding reciprocal hemizygosity tests, we agree that they would provide a valuable means of assessing the evolutionary contribution of candidate genes. However, generating the necessary reagents in *D. simulans* represents a substantial undertaking beyond the scope of the present study, particularly given the expected modest effects of individual loci underlying this highly polygenic trait. We have nevertheless revised the end of the Results section to mention reciprocal hemizygosity tests as an important direction for future work.

**Reviewer #2 (Recommendations for the authors):**

(1) Provide more details about the selection tests: which sequences were used? Please report p-values. It would also be important to discuss the possibility of false positives caused by a bottleneck in D. sechellia. A genome-wide analysis could help to see if the bottleneck increased the signal of positive selection.

We are not entirely sure what additional analyses are being suggested. The sequences and methods used for the selection analyses are described in the Methods, and the statistical support for these analyses is reported in the Supplementary Material (MK test p-values and FUBAR posterior probabilities). We are also unclear as to how a genome-wide analysis would address the interpretation of the gene-specific selection analyses presented here. If the reviewer intended a different analysis, we would appreciate further clarification.

(2) The significance level of the CMH test needs to be determined with the effective population size, not with the census size, as done by the authors. This is important, as it is not clear if the candidate genes remain significant after significance adjustment based on the effective population size.

We thank the reviewer for this comment. In our analyses, effective population sizes were explicitly incorporated by Bait-ER to model the effects of genetic drift. The CMH significance thresholds, however, were obtained from neutral forward simulations following the recommended workflow for the method, which requires census population sizes rather than effective population sizes as input. To make these simulations as realistic as possible, we therefore used the observed census population sizes at each generation of the experimental evolution. Estimating generation-specific effective population sizes for use in an alternative simulation framework would require substantially more temporal data than are available here (e.g., sequencing many additional time points) and is beyond the scope of the present study.

(3) Include the allele frequency trajectory across time for the candidate genes.

We thank the reviewer for this suggestion. However, plotting allele frequency trajectories for the candidate genes is not straightforward because the evolve-and-resequence analysis identified broad linked genomic regions rather than individual causal variants. Each candidate region contains numerous SNPs spanning several kilobases, with each SNP exhibiting its own allele frequency trajectory across the 10 replicate populations. Consequently, there is no single representative trajectory for a given candidate gene, and plotting all SNPs within each region would be difficult to interpret. For *kraken*, however, we identified seven candidate regulatory SNPs and have plotted their individual allele frequency trajectories, which we include in Author response image 1 to illustrate the diverse patterns observed:

**Author response image 1.**

(4) Estimate the effect of candidate loci in the GWAS data (independent of significance).

We thank the reviewer for this suggestion. However, we are not entirely sure what analysis is being proposed. In particular, it is unclear which variants the reviewer is referring to, as our candidate genes are associated with multiple linked variants rather than a single causal SNP. Consequently, we are unsure how the effect of a candidate locus should be estimated in the GWAS dataset. Moreover, there is no guarantee that the same variants are represented in both datasets. For example, variants filtered out during the GWAS because of low allele frequency may subsequently have increased in frequency during experimental evolution and therefore contributed to the evolve-and-resequence signals. We would appreciate further clarification of the analysis the reviewer has in mind.

(5) Discuss the challenge of false positives (see: 10.1016/j.cub.2020.12.023).

We thank the reviewer for this suggestion. We agree that false positives (as well as false negatives) are an important consideration when studying complex polygenic traits, both through the initial “screening” efforts (e.g., GWAS, experimental evolution) and follow-up functional validation (e.g., RNAi, mutant, overexpression analyses). We believe this issue is already addressed in the manuscript through our discussion of the limitations of the individual approaches, the polygenic nature of OA resistance, and our cautious interpretation of the functional validation results. Our conclusions are limited to identifying candidate genes that contribute to OA resistance, rather than claiming to have identified all of the loci or variants underlying the evolution of this trait. We are therefore not sure what additional discussion the reviewer has in mind and would appreciate further clarification if a specific point from the cited study is intended.

(6) The figures with the Manhattan plots should be improved to indicate the overlapping genes in the Manhattan plots, rather than in the circle figures below. By using different colors this should be quite easy and clean.

We thank the reviewer for this suggestion. However, we believe Author response image 2 more appropriately illustrate the overlap between the CMH and BaitER analyses. Although the Manhattan plots display individual SNPs, our candidate loci are defined by broader genomic regions comprising blocks of linked significant SNPs rather than by single variants. Simply highlighting SNPs within overlapping regions would therefore add visual complexity without providing additional biological insight beyond that already captured by the circos plots. As an illustration, we provide here an example of the generation 50 Manhattan plot with overlapping SNPs highlighted in red:

**Author response image 2.**

(7) A more focused discussion of the assumption that functional data from *D. melanogaster* can explain resistance in D. simulans or D. sechellia. At some places, epistatic interactions are mentioned, but the reviewer feels that the entire screen is based on the idea that similar effects are found across species, hence, this needs to be adequately reflected in the discussion.

We thank the reviewer for raising this important point. We would like to emphasize that our study was not based on the assumption that functional effects identified in *D. melanogaster* or *D. simulans* necessarily explain the evolution of OA resistance in *D. sechellia*. Rather, our motivation stemmed from the longstanding difficulty of identifying individual genes underlying this highly polygenic trait using mapping approaches alone. We therefore sought to combine orthogonal experimental approaches to identify genes contributing to OA susceptibility (of *D. melanogaster* and *D. simulans*) and resistance (*D. sechellia*). We hoped, but did not assume, that genes supported by multiple independent lines of evidence would be informative for understanding the natural evolution of OA resistance in *D. sechellia*. Indeed, we acknowledge in the manuscript that the cell-based CRISPR screen, experimental evolution, and the natural evolution of *D. sechellia* occurred under very different selective contexts and timescales. As reflected in the title of our manuscript, our conclusions are intentionally framed around the identification of novel toxin resistance loci, while remaining cautious about their evolutionary interpretation.

(8) Discuss that the controls in the RNAi test were quite variable. Could this reflect some problems with the assay?

We do not believe that the variability among the control lines reflects a problem with the assay. Rather, the different controls (Gal4, UAS-RNAi etc.) represent distinct genetic backgrounds, each of which may exhibit a different baseline level of OA resistance. Indeed, in our recent GWAS of OA resistance (doi:10.1093/g3journal/jkag032), we observed substantial natural variation in OA resistance among *D. melanogaster* and *D. simulans* lines. Importantly, each RNAi line was compared with its corresponding genetic background control, so differences among control lines do not affect the interpretation of the individual RNAi experiments.
